# The versatility of the CHASE domain in one-component systems of c-di-GMP signal transduction from *P. aeruginosa*

**DOI:** 10.1128/jb.00370-25

**Published:** 2026-05-28

**Authors:** Simone Angeli, Chiara Scribani-Rossi, Giulio Colasanto, Sharon Spizzichino, Serena Rosignoli, Alessandra Giorgi, Francesca Cutruzzolà, Alessio Paone, Angela Tramonti, Roberto Contestabile, Robert Montoya, George A. O'Toole, Alessandro Paiardini, Serena Rinaldo

**Affiliations:** 1 Department of Biochemical Sciences A. Rossi Fanelli, Sapienza University of Rome 9311, Rome, Italy; 2 Centre for Regenerative Medicine “Stefano Ferrari”, Department of Life Sciences, University of Modena and Reggio Emilia 9306, Modena, Italy; 3 Istituto di Biologia e Patologia Molecolari, Consiglio Nazionale delle Ricerche 9327, Rome, Italy; 4 Geisel School of Medicine at Dartmouth 12285 , Hanover, New Hampshire, USA; Indian Institute of Technology Bombay, Mumbai, Maharashtra, India

**Keywords:** heme, biofilm, *Pseudomonas aeruginosa*, c-di-GMP, copper

## Abstract

Cyclic di-GMP (c-di-GMP) is a key bacterial second messenger that regulates a wide range of cellular processes, including biofilm formation and virulence. Multi-domain one-component systems regulate c-di-GMP synthesis and turnover in response to external signals. Periplasmic sensing of environmental cues is performed by versatile but conserved sensory domains, including members of the CHASE4 superfamily. Here, we explore the promiscuity of the CHASE4 domain in c-di-GMP signal transduction in *Pseudomonas aeruginosa* by analyzing two CHASE4-containing transducers from the virulent PA14 strain, namely PA14_53310 and PA14_37690. With the integration of biochemical and biophysical methods, such as UV-Vis spectroscopy, circular dichroism, and isothermal titration calorimetry, we demonstrate that the CHASE4 domains of these proteins possess different ligand specificities. We show that PA14_53310 is a diguanylate cyclase, and that its enzyme activity is controlled by heme binding to its periplasmic CHASE4 domain. The PA14_37690 CHASE4 domain, on the other hand, does not bind heme, but likely recognizes copper, indicating a role in metal ion sensing. A comparison with the PAO1 counterpart, that is, PA0847 and PA2072, is discussed and the divergences highlighted. This work demonstrates that the CHASE4 domain is a multifunctional sensory module that can be calibrated to detect a range of environmental cues, thus providing a mechanism for c-di-GMP signaling refinement in *P. aeruginosa*. These results shed light on the molecular basis for the functional diversification of CHASE4-containing one-component systems and their role in bacterial adaptation.

**IMPORTANCE:**

The results presented in this study reveal a fascinating versatility of the CHASE4 domain in PA14 *Pseudomonas aeruginosa* cyclic di-GMP signaling systems (i.e., PA14_53310 and PA14_37690), highlighting its ability to sense different environmental cues, such as heme and copper. In the context of infection, the interplay between heme and copper sensing could be particularly relevant, being both nutrients or poisons. Their sensing (and adaptive response) would be a significant advantage for the bacterium, allowing it to optimize its virulence and survival strategies. Therefore, these results represent the starting point for future *in vivo* studies aimed at exploring the relevance of heme and copper in pathogenesis, to finally develop novel anti-virulence strategies targeting the pathogen’s ability to adapt to the host environment.

## INTRODUCTION

Among the diverse array of signaling molecules employed by bacteria, the second messenger cyclic diguanylate (cyclic di-GMP [c-di-GMP]) is a central regulator of key metabolic reprogramming events, including the planktonic-to-sessile transitions, the formation and maintenance of biofilm, and its dispersion (1). The mature biofilm is a metabolically active and multifaceted ecosystem where bacteria can survive and adapt in a fluctuating environment, as in the case of chronic infection sites (2). Biofilm metabolism and maintenance depend on intricate signal transduction networks that perceive external stimuli and elicit appropriate cellular responses, which in turn lead to the variation of intracellular levels of c-di-GMP (3). The intracellular concentration of c-di-GMP is finely controlled by the opposing activities of diguanylate cyclases (DGCs), which synthesize the second messenger from two GTP molecules, and phosphodiesterases (PDEs), which are responsible for its degradation. DGC and PDE activities are associated with the conserved GGDEF and EAL domains, found, sometimes in tandem, at the C-terminus of c-di-GMP-related transducers (4). The redundancy of genes encoding GGDEF and/or EAL, and the fine-tuning of the corresponding transducers, in terms of both turnover and cellular localization, allows bacteria to integrate a multitude of environmental cues, ranging from nutrient availability to the presence of host factors. The downstream response, which includes c-di-GMP sensing (5), leads to the control of complex processes such as virulence, cell cycle progression, and biofilm formation (2, 3).

*Pseudomonas aeruginosa* is an opportunistic gram-negative pathogen, renowned for its metabolic versatility and capability of resilience in biofilm structures difficult to be eradicated (6–8). This bacterium represents one of the most important model systems for studying biofilm, given its biomedical relevance: it is a major cause of severe nosocomial infections, particularly in immunocompromised individuals and those with cystic fibrosis, where its ability to form antibiotic-tolerant biofilms poses a significant clinical challenge (9). The complexity of its c-di-GMP network is underscored by the presence of over 40 proteins predicted to be involved in c-di-GMP turnover, a redundancy that suggests a high degree of specialization and the need to respond to a vast spectrum of environmental signals (10, 11).

The diversity within the *P. aeruginosa* species itself adds another layer of complexity. The two most widely studied strains, PAO1 and PA14 (with the former being the lab strain historically studied), exhibit significant differences in their virulence and genomic content. PAO1, a well-characterized laboratory-adapted strain, is considered moderately virulent. In contrast, PA14, a clinical isolate from a burn wound, is a hyper-virulent strain that shows enhanced pathogenicity across a broad range of hosts (12). These differences include a unique pathogenicity island in the PA14 genome, different virulence factors, and a subset of specific genes transcriptionally regulated in infection models (12–14).

The specificity of the c-di-GMP signaling pathways in *P. aeruginosa* is largely conferred by sensory domains that are typically fused to the DGC or PDE catalytic domains. One such key sensory module found in the GGDEF-EAL proteins is the CHASE4 domain, which is predicted in the periplasmic portion of transmembrane transducers to detect small molecules in the extracellular *milieu*. In PAO1, the CHASE4 domain has been found in the PA0847 and PA2072 genes (10). A recent work identified the structural and functional determinants of these two CHASE4 domain activations. The CHASE4 domain of the protein PA0847 has been shown to bind ferric iron, a crucial nutrient, thereby influencing bacterial motility (15, 16); on the other hand, the CHASE4 domain from PA2072 responds to ferric iron but also potentially to heme, to ultimately control the DGC activity of another GGDEF protein (named PA1851 or ImcA) via protein-protein interaction (15). While the effect of iron in disrupting this interaction (and therefore DGC inhibition) has been functionally analyzed, the potential role of heme is only mentioned. Given the iron-dependent readout of PA2072, the authors named this transducer IsmP, Iron-sensing membrane protein. These findings establish a role for CHASE4 domains as important sensors of metal ions and porphyrins in the PAO1 strain.

However, given the significant genomic and phenotypic differences between the PAO1 and PA14 strains, it is plausible that the functions of their homologous signaling proteins have diverged to suit their distinct lifestyles. While the roles of PA0847 and PA2072 are beginning to be understood in PAO1, the functions of their respective homologs in the hyper-virulent PA14 strain, PA14_53310 and PA14_37690, have remained unexplored. In this study, we address this knowledge gap by performing a detailed biochemical and biophysical characterization of these two PA14 proteins. We hypothesized that the ligand specificities and regulatory outputs of these signaling systems would differ between the two strains, reflecting their adaptation to different environmental niches and pathogenic strategies.

This work sheds new light on how *P. aeruginosa* fine-tunes its response to environmental cues, providing a deeper understanding of the molecular mechanisms that underpin its adaptability and virulence.

## RESULTS

### Isolation and characterization of PA14_53310

**Fig 1:**
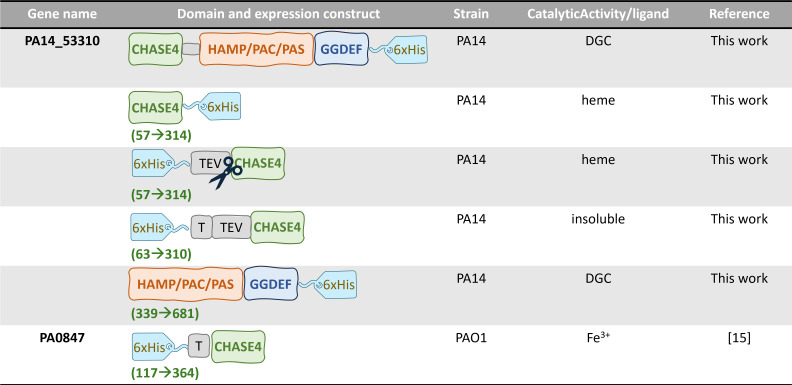
Domain organization of the proteins used in this study derived from PA14_53310. All the expression constructs (including the full-length and the truncated versions) are summarized in the figure as a cartoon, including the affinity tag (6×His) and the residue range selected for the truncated versions. The strain, the observed catalytic activity, and ligand are also reported. Proteins belonging to the study of reference 15 are also depicted for rapid comparison (rows labeled with ref). TEV: cleavage site for TEV protease; T: cleavage site for thrombin; scissors indicate that the protein has been characterized after His-tag removal. Residues encompassing PA0847 CHASE4 domain have been inferred from oligonucleotides sequence used for subcloning (15). The same cartoon is used to depict the expression construct in the subsequent figures, to allow the reader to rapidly identify the construct described in a specific figure.

**Fig 2:**
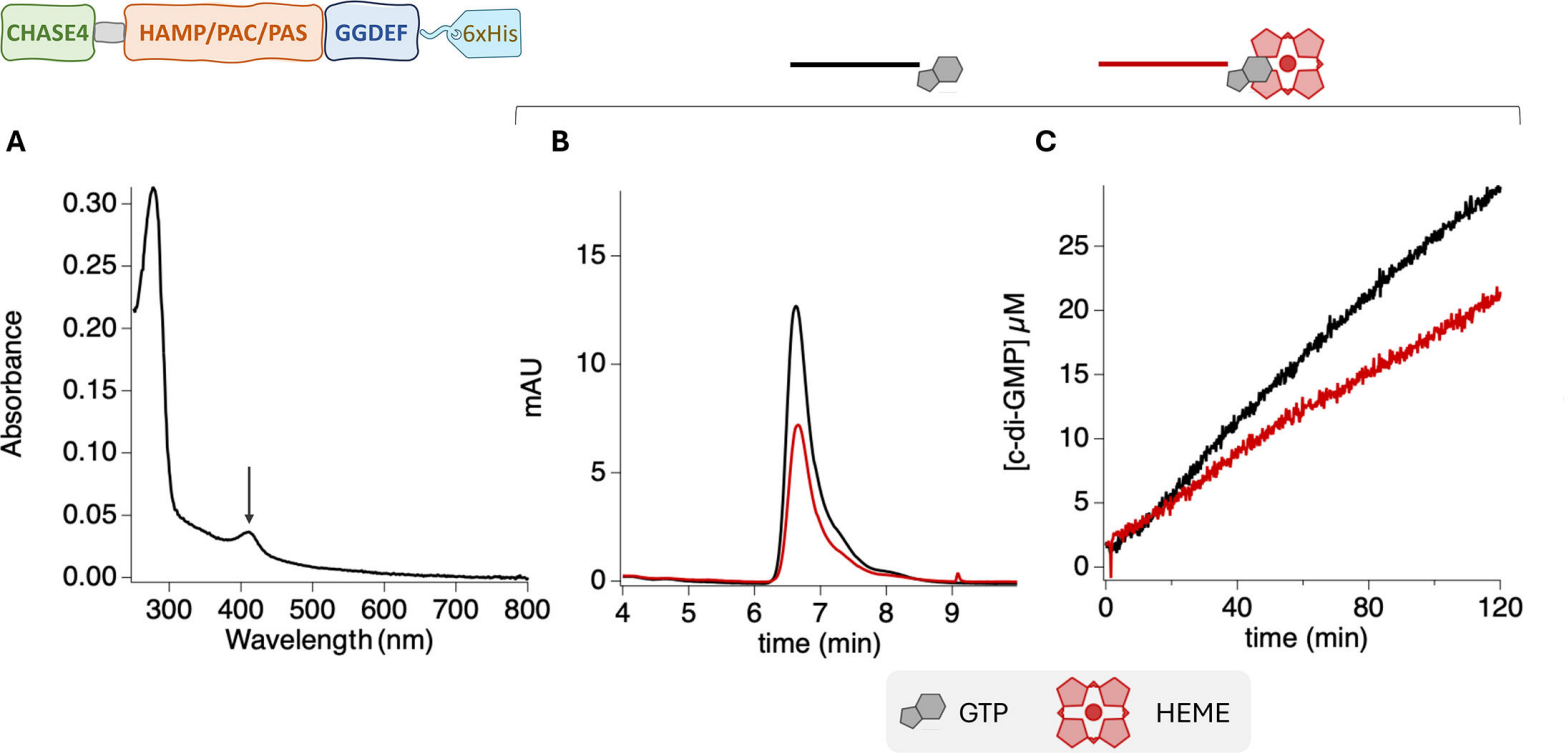
Characterization of PA14_53310. (**A**) UV-Vis spectrum of purified PA14_53310. The arrow indicates the observed peak in the visible region of the spectrum. (**B**) DGC assay with 2 µM PA14_53310 solution incubated with manganese and 100 µM GTP for 120 min, with or without pre-incubation with 10 µM heme solution (red and black lines, respectively); c-di-GMP content, reported in the figure, was evaluated by RP-HPLC. (**C**) DGC continuous assay carried out following the CD signal of c-di-GMP at 285 nm according to reference 20; in the figure, the signal has been converted into µM c-di-GMP using the calibration curve extrapolated from the reference spectra reported in Fig. S2B. Kinetics were run with or without pre-incubation with 10 µM heme solution (red and black lines, respectively). A small hysteretic trend is observed, as expected for DGC (19); no product inhibition is observed in this timeframe. Linear fit yielded an observed v_0_ = 0.25 ± 0.01 min^−1^ and a v_0_ = 0.18 ± 0.01 min^−1^, without or with heme, respectively.

Full-length PA14_53310 was expressed as a C-terminal His-tagged construct (Fig. 1) and extracted with DDM detergent (Fig. S1A); protein identity was verified by mass spectrometry (data not shown). Nanodisc assembly, although successful, yielded a largely inactive protein (Fig. S1B through E), and therefore kinetics and biochemical characterization were carried out on purified protein extracted with the DDM detergent. As shown in Fig. 2A, the UV-Vis spectrum of the protein shows a small peak in the visible region (411 nm), and it is active as DGC (Fig. 2B, black traces). As previously published by reference 17, two complementary approaches have been used to evaluate DGC turnover: (i) a discontinuous assay via RP-HPLC, based on nucleotide separation (left panel in Fig. 2B), where the reaction is stopped at different times and then analyzed by independent runs (Fig. S2A); (ii) a continuous assay (right panel in Fig. 2B), where c-di-GMP is quantified by taking advantage of its specific circular dichroism (CD) signal (Fig. S2B). Both techniques confirm that the protein is active as DGC and that no appreciable feedback inhibition is observed in this timeframe. A protein construct including the sole cytoplasmic portion of PA14_53310 confirms that DGC activity (Fig. S2C). Moreover, although subtle, a lag phase could be appreciated in the continuous assay, which is indicative of hysteretic kinetics required to populate the catalytically competent dimeric active site (18, 19).

The absorbance peak in the visible region of the spectrum is compatible with a (very diluted) Soret signal, typical of heme proteins. To probe whether the heme cofactor could participate in the one-component transduction performed by PA14_53110, the DGC assay was repeated by pre-incubating the protein with excess heme. As shown in Fig. 2B, red traces, the co-presence of heme leads to a reduction of the observed turnover of ~1.4-fold. Given the involvement of heme in controlling catalysis, we tested heme binding to PA14_53110 by spectroscopy; the titration was performed in a detergent-based buffer (DDM), and a parallel “blank” titration with the sole heme has been done for data analysis. This approach is crucial since free heme UV-Vis spectrum is highly sensitive to pH or buffer components. UV-Vis spectra suggest that PA14_53110 binds to heme (Fig. S3A and B); it should be mentioned that, contrary to expectations, protein-free heme spectra show an expected Soret peak at 404 nm (a pronounced shoulder below 400 nm is expected in a water-based buffer, Fig. S3C). Nevertheless, DDM only affects the spectroscopic signature of free heme, while that of heme-iron coordination in well-known heme proteins is not significantly affected (Fig. S3D).

### Isolation and characterization of PA14_53310 CHASE4 domain

To better characterize the heme-binding properties, the biochemical characterization was next performed on water-soluble isolated domains. The catalytic activity of PA14_53310 is tuned by heme, a metabolite relevant to both intracellular homeostasis and environmental perception. Indeed, GGDEF (EAL) one-component transducers can be tuned by both extracellular and intracellular signals, as in the case of RmcA from *P. aeruginosa*. The periplasmic Venus Fly Trap (VFT) and the cytoplasmic PAS domains of RmcA respond to extracellular arginine and FADH_2_ (as a readout of the intracellular reducing power), respectively, thus leading to a multicompartment fine-tuning of RmcA-dependent c-di-GMP level (21). Therefore, the identification of the domain(s) involved in environmental sensing can be complex in this kind of multidomain protein.

In the case of PA14_53310, both the periplasmic CHASE4 domain and the cytoplasmic PAS/PAC domain are eligible to be the heme-sensing domain. We focused our analysis on the CHASE4 domain, considering recently published data on the PAO1 counterpart protein, that is, PA0847. In this recent report, the authors showed that the CHASE4 domain, thought to be localized to the periplasmic space, binds to ferric iron with a K_D_ = 16 µM (15); nevertheless, the authors also proposed that the homologous CHASE4 domain from the PA2072 transducer responds to both ferric iron (K_D_ = 7 µM) and heme (15). If this versatility is also true for the PA14_53310 CHASE4 domain, it is likely that the heme-dependent DGC tuning observed with the full-length protein involves the CHASE4-heme interaction.

**Fig 3:**
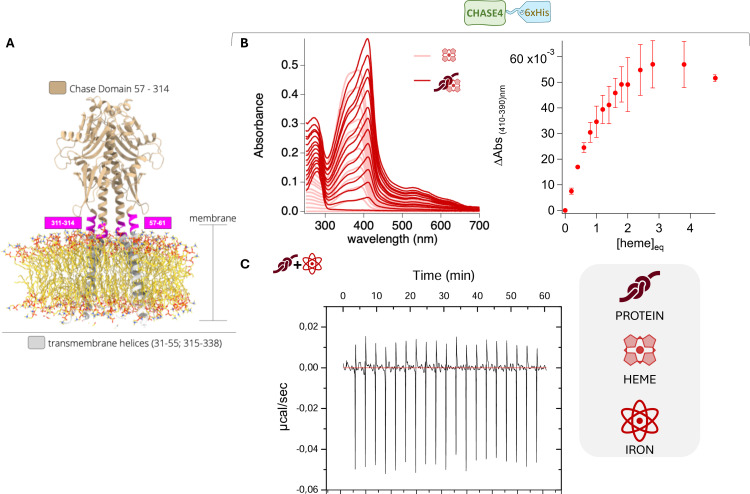
Characterization of the PA14_53310 CHASE4 domain. (**A**) Schematic topology of PA14_53310. Two predicted transmembrane helices (gray) insert into the membrane, placing the CHASE4 domain outside the cytoplasm. Domain scanning and modeling place CHASE4 at residues 57–314 (light sepia brown plus magenta). This span is highlighted in the cartoon; extra residues in magenta were included in this work and do not superpose with those selected by reference 15 to isolate the CHASE4 domain from PA0847. Downstream domains (PAS and GGDEF) begin after residue 314 and are not depicted. Predicted membrane helices and CHASE4 boundaries are based on TMHMM and Pfam/AlphaFold2 analyses. (**B**) UV-Vis spectra (left panel) and analysis (right panel) of 4.6 µM PA14_53310 CHASE4 in the presence of increasing amounts of hemin (red lines); equivalents have been calculated considering the monomeric concentration of the protein. For comparison, the corresponding hemin spectra are also included (pink lines). Spectral analysis reported in panel B has been done by plotting the variation at 410 nm minus the contribution at 390 nm (representative of non-coordinated heme iron), as a function of heme equivalents. Data are the average of two independent experiments ± SD. The linear trend is compatible with a titration behavior; the deviation at two equivalents should indicate protein saturation. (**C**) Isothermal titration calorimetry (ITC) experiment carried out by titrating 5 µM PA14_53310 CHASE4 with FeCl_3_, showing the recorded heat variation upon ligand addition over time. Lack of sigmoidal trend indicates that there is no binding under these experimental conditions.

PA0847 and PA14_53310 show ~100% sequence identity, except for an extra 54-residue N-terminal peptide only found in PA0847 and two conservative substitutions (Fig. S4). Homology modeling allowed us to define the boundaries of the PA14_53310 CHASE4 domain (residues 57–314; Fig. 3A). This prediction includes seven extra residues (highlighted in magenta in Fig. 3A), compared to the PAO1 PA0847 CHASE4 domain previously characterized (15).

Expression and purification of the PA14_53310 CHASE4 domain yielded a homogeneous sample further characterized in this study (Fig. S5A).

Heme titration of the PA14_53310 CHASE4 domain shows a typical heme iron (five) coordination profile with a maximum at 410 nm (Fig. 3B, left panel), and saturation occurring in the presence of two heme equivalents (Fig. 3B, right panel). To further verify the specificity of binding, the dissociation constant was measured according to (22). The slow kinetics yielded a k_off1_ = 1.5 ± 0.6 × 10^−5^ s^−1^ and k_off2_ = 5.5 ± 2.1 × 10^−4^ s^−1^, in agreement with the values observed for other heme-based sensors (22) (Fig. S5B).

Although with poor resolution due to low yield, heme binding was also observed in the His-tag-free protein variant (Fig. S6A); a construct designed according to the previously published PAO1 PA0847 CHASE4 variant (as an N-terminal His-tagged protein) was expressed unsuccessfully (Fig. S6B). Surprisingly, the PA14_53310 CHASE4 domain does not bind to ferric iron, contrary to the previously published PAO1 counterpart (Fig. 3C). We conclude that PA14_53310 is a one-component system working as a DGC, able to tune its catalytic activity via the periplasmic CHASE4 domain in response to heme. In this study, only b-type heme was assayed.

### Isolation and characterization of PA14_37690

**Fig 4:**
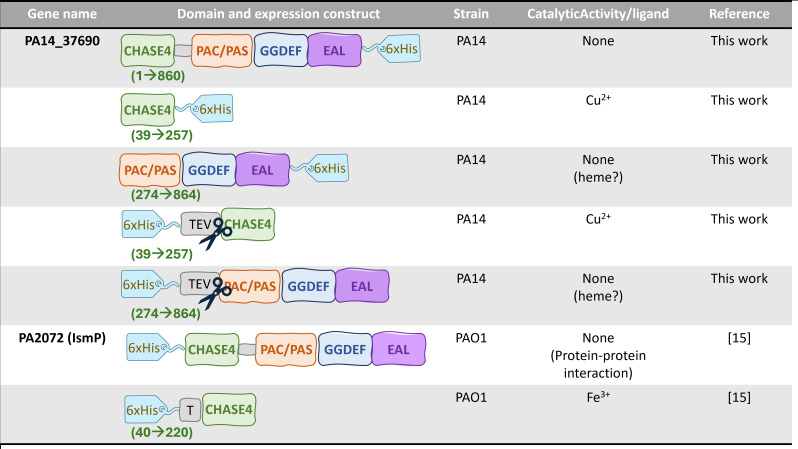
Domain organization of the proteins used in this study derived from PA14_37690. All the expression constructs (including the full-length and the truncated versions) are summarized in the figure as a cartoon, including the affinity tag (6×His) and the residue ranges selected for the truncated versions. The strain, the observed catalytic activity, and the ligand are also reported. Proteins belonging to the study of reference 15 are also depicted for rapid comparison (rows labeled with ref). TEV: cleavage site for TEV protease; T: cleavage site for thrombin; scissors indicate that the protein was characterized after His-tag removal. Residues encompassing the IsmP CHASE4 domain were inferred from the oligonucleotide sequences used for the subcloning (15).

PA14_37690 is homologous to PAO1 PA2072 (IsmP) protein, a membrane transducer controlling protein-protein interactions via its CHASE4 periplasmic domain, responding to ferric iron and possibly to heme (15). The small sequence differences between the two homologs are highlighted in Fig. S7A. The constructs characterized in the present work are summarized in Fig. 4. Full-length PA14_37690 has been detergent-extracted and purified with good yield (Fig. S7B); the UV-Vis spectrum of the purified protein shows a shoulder above 400 nm (Fig. S7C), suggestive of heme or a cofactor showing a peak in that visible region of the spectrum. No DGC or PDE activity has been observed (Fig. S8A, black traces), in agreement with previously published data on IsmP (15). We tested heme binding spectroscopically, also considering the aforementioned DDM-related issue with the reference spectra. As depicted in Fig. S8B, the heme-containing protein sample shows a different Soret peak compared with free heme (408 nm vs 404 nm for the corresponding free heme; red and pink traces, respectively). Contrary to PA14_53310, excess heme does not have any impact on the catalytic assay readout (Fig. S8A, red traces).

### Isolation and characterization of PA14_37690 CHASE4 domain

As for PA14_53310, PA14_37690 has two possible domains potentially able to bind to heme, that is, the periplasmic CHASE4 and the cytoplasmic PAS domain. Homology modeling data identified the CHASE4 domain between residues 39 and 257 of PA14_37690 (Fig. S7A), while the cytoplasmic portion starts from residue 274 to the C-terminus (Fig. 4).

**Fig 5:**
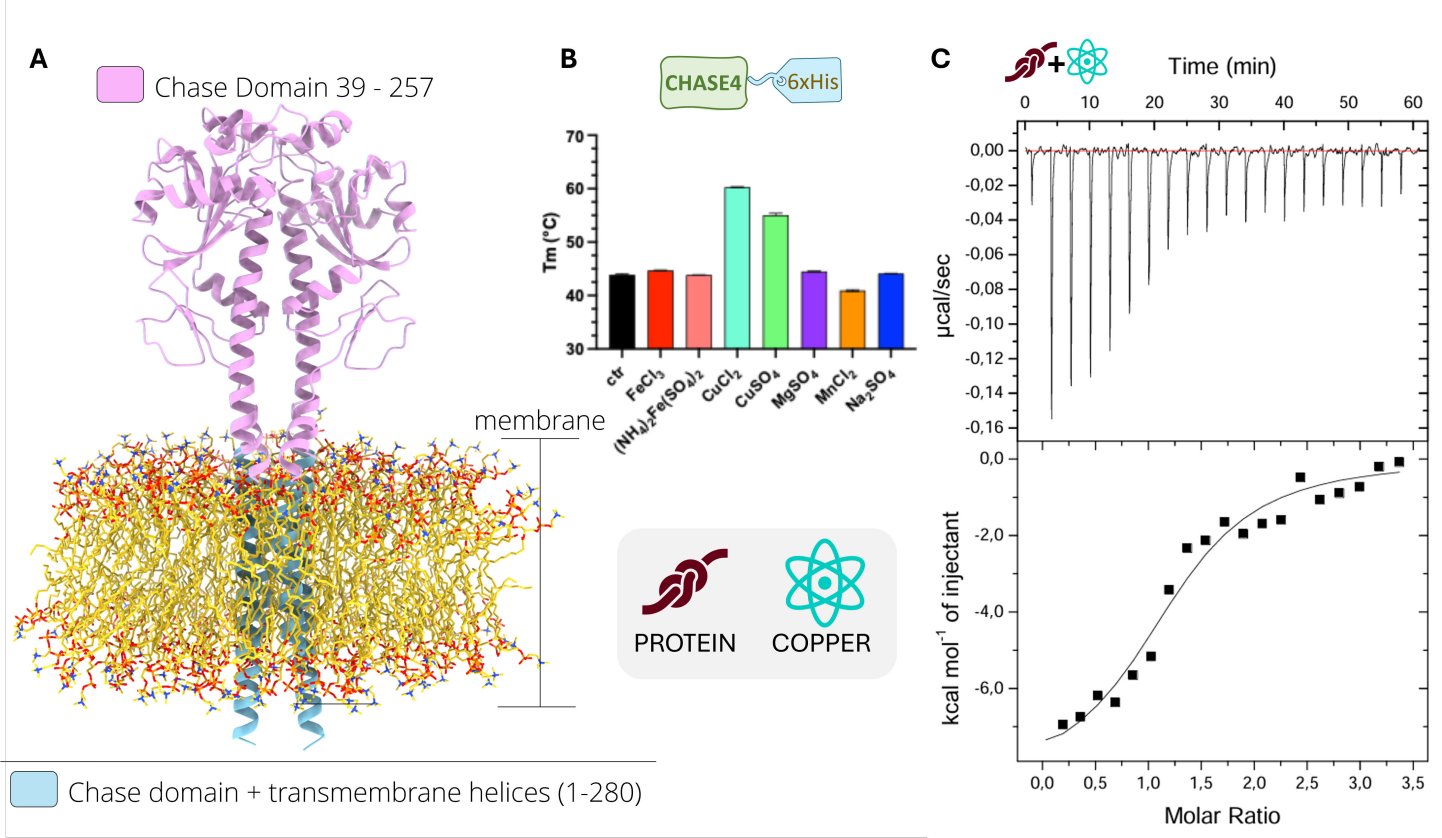
Characterization of PA14_37690 CHASE4 domain. (**A**) Schematic topology of *P. aeruginosa* PA14_37690. Two N-terminal transmembrane helices (cyan cylinders) anchor the protein in the inner membrane. The periplasmic CHASE4 sensor domain occupies residues 39–257, as identified by Pfam/InterPro and confirmed by the PA2072 structure (PDB: 8Z84). Residue numbering and domain boundaries are indicated. The cytoplasmic C-terminal region contains PAS/PAC, GGDEF, and EAL domains (not shown) beyond residue 280. (**B**) Fluorescence change, expressed as fractional variation, as a function of temperature of 4 µM PA14_37690 CHASE4 domain in the presence of the indicated salts (70 µM). The curves depicted in Fig. S11C were fitted to the Boltzmann equation to obtain the melting temperatures represented in the histogram. (**C**) ITC experiment carried out by titrating PA14_37690 CHASE4 with CuSO_4_; in the upper panel, the recorded heat variation upon ligand addition over time, and in the lower panel, the calculated heat variation per mol of injectant for each peak. The sigmoidal trend was fitted with the one-site model (MicroCal Origin), yielding the thermodynamic signature reported in Table S1.

The PA14_37690 CHASE4 domain, whose model is depicted in Fig. 5A, was purified to homogeneity in a good yield (Fig. S9A). Heme binding to CHASE4 seems to be spurious (Fig. S9B), considering the very similar spectroscopic profile to the RmcA VTF heme titration (His-tagged, which does not bind to heme) (Fig. S9C).

On the other hand, the cytoplasmic portion was found to be poorly expressed (Fig. S10A); nevertheless, the spectroscopic changes observed upon heme titration are appreciable and compatible with a mixed species, the classic peaking at 410 nm and a non-histidine five-coordinate species, with a pronounced shoulder encompassing the 370–3390 nm range (Fig. S10B) (23–25). The His-tag-free construct shows the same poor expression and comparable spectroscopic changes upon heme titration (Fig. S10C).

Given the low yield of this protein and the absence of a functional readout, a more rigorous characterization was not possible, and, at this stage, any further consideration could be overly speculative.

IsmP CHASE4 (whose sequence is highlighted in green in Fig. S7) was found to bind ferric iron in solution with a K_D_ = 7 µM (15). PA14_37690 CHASE4 (whose sequence is highlighted in magenta in Fig. S7) does not bind to ferric iron, showing a non-binding ITC profile (Fig. S11A), close to that observed in the control experiment (i.e., ferric iron vs buffer, Fig. S11B). Given the high similarity between the two proteins, we screened other metals to unveil a possible alternative metal-binding activity. The screening was performed by evaluating the effect of different metals on the thermal stability of the protein by differential scanning fluorimetry (DSF). As depicted in Fig. 5B (and Fig. S11C), cupric salts lead to a dramatic shift in the melting temperature of the protein. The binding was quantitatively verified by ITC, yielding a classical one-site binding model (Fig. 5C) and the thermodynamic signature reported in Table SI. As compared to IsmP, PA14_37690 CHASE4 contains histidine 39, absent in the PAO1 truncated version. Site-directed mutation indeed affects copper binding in terms of mechanism, leading to the loss of the enthalpy contribution, which is entropically compensated (Fig. S10D and Table SI). These data suggest that histidine 39 likely locally contributes to the copper coordination geometry occurring upon metal binding to the PA14_37690 CHASE4. When the effect of copper on thermal stability is assayed on the full-length protein (by CD, since the DSF in detergent is negatively affected), a thermal shift toward lower temperature is observed (Fig. S12A). This apparent opposite result, as compared to the isolated CHASE4 domain, is not so surprising considering that the full-length protein has a multidomain architecture, and the CD signal evaluates the overall secondary structure; regardless of the direction of the thermal effect, PA14_37690 binds copper with a 1:1 stoichiometry through the CHASE4 domain, and the presence of copper triggers a conformational rearrangement, which likely involves downstream domain(s). Copper binding has been further confirmed in the His-tag-free version of the CHASE4 construct (Fig. S12B).

### Impacts of deletion of the *PA14_53310* and *PA14_37690* genes on biofilm and swarming motility

In light of the biochemical characterization reported above, and considering the possible differences between PAO1 and PA14 strains, the impact of PA14_53310 and PA14_37690 gene deletion was assayed by looking at the sessile lifestyle and biofilm-related phenotypes, including swarming motility, c-di-GMP, and exopolysaccharides (EPS) production.

**Fig 6:**
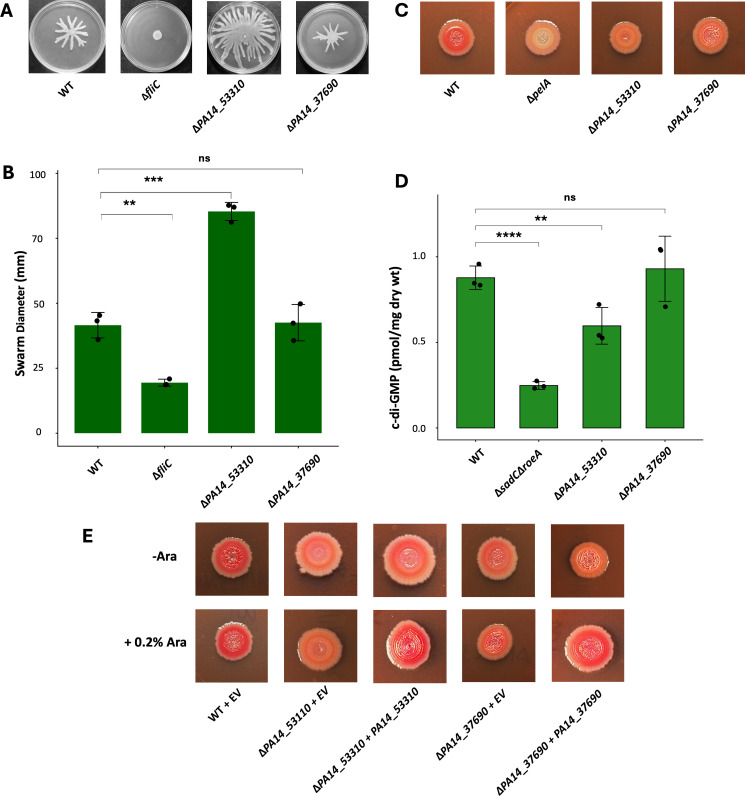
Phenotypic characterization of the ∆*PA14_37690* and *∆PA14_53310* mutants. (**A**) Representative swarm images of the indicated strains grown on M8 agar swarm plates for 16 h. (**B**) Graph showing the average diameter (in millimeters) of swarms from triplicate analysis of each strain. Analyzed by ANOVA with Tukey’s post-test comparison, **P < 0.01 and ***P < 0.001. (**C**) Representative CR images of the indicated strains cultures on M8 plates solidified with 1% agar, incubated for 16 h at 37°C followed by 4 days at room temperature. (**D**) Quantification of c-di-GMP levels in the indicated strains grown on M8 swarm plates for 16 h prior to harvest for extraction of nucleotides and measurement of c-di-GMP levels via mass spectrometry. Performed in triplicate with three technical replicates per strain. Analyzed by ANOVA with Tukey’s post-test comparison, **P < 0.01, ****P < 0.0001, and ns, not significant. (**E**) Representative Congo red binding images showing complementation of the ∆*PA14_37690* and *∆PA14_53310* mutants by expression of the respective genes *in trans* from an arabinose-inducible plasmid pMQ72. WT and deletion mutants carrying the empty vector (EV, pMQ72) were included as controls.

Swarming motility has been linked to cyclic-di-GMP level and has been well documented in *P. aeruginosa* PA14 (26, 27). This mode of motility enables coordinated movement across semisolid surfaces and requires both a functional flagellum and production of rhamnolipid surfactants. A mutant that lacks a functional flagellum (∆*fliC*) and is therefore unable to swarm was used as a negative control. A mutation in the *PA14_53310* gene shows a hyperswarming phenotype (i.e., an increase in surface coverage) compared to the wild-type strain. In contrast, the ∆*PA14_37690* mutant displayed swarming comparable to wild type (Fig. 6A and B).

Extracellular matrix production is typically associated with biofilm formation and relies on EPS as a key structural component. For *P. aeruginosa* PA14, the EPS called Pel has been identified as a key contributor to biofilm development (28) and has been positively associated with levels of c-di-GMP (29). To evaluate the effects of deletion of the *PA14_53310* and *PA14_37690* genes on Pel production, Congo red (CR) assays were performed. CR is commonly used to identify mutants defective in the production of Pel (30). A Pel-deficient mutant (∆*pelA*) fails to bind CR (31), resulting in the production of smooth, white colonies, and acts as the negative control (Fig. 6C). Both ∆*PA14_53310* and ∆*PA14_37690* mutants showed diminished CR binding compared to the wild-type strain, although these strains showed more CR binding than the ∆*pelA* negative control (Fig. 6C). To determine whether the reduced CR binding observed in these mutants was associated with altered global levels of c-di-GMP, we quantified levels of this second messenger for surface-grown cells using mass spectrometry, as reported (26). The ∆*PA14_53310* mutant exhibited a significant reduction in c-di-GMP levels relative to the WT, whereas the ∆*PA14_37690* mutant showed no change in c-di-GMP levels (Fig. 6D), in line with the swarming and *in vitro* phenotypes. The ∆*sadCroeA* mutant serves as a control for a strain with reduced levels of c-di-GMP compared to the wild type (27).

### Complementation of the ∆*PA14_53310* and ∆*PA14_37690* mutant strains

To confirm the CR binding phenotypes observed in the ∆*PA14_37690* and ∆*PA14_53310* mutants are due to specific disruption of these genes, complementation experiments were performed by expressing each gene *in trans* from a plasmid (pMQ72) under the control of an arabinose-inducible promoter (Fig. 6E). As observed above, both ∆*PA14_37690* and ∆*PA14_53310* mutants displayed reduced CR binding relative to the wild-type strain. Introduction of the plasmids carrying PA14_37690 or PA14_53310 into their respective deletion mutants restored Congo red binding levels comparable to WT when the expression of the genes on these plasmids was induced by arabinose. The empty vector (EV) control pMQ78 showed no complementation, as expected (Fig. 6E). These results indicate that the loss of PA14_37690 and PA14_53310 contributes to EPS production as assessed by CR binding.

## DISCUSSION

Biofilm formation and maintenance are dynamic processes requiring constant environmental scanning to reprogram community metabolism and architecture in response to surrounding conditions (2). This adaptive response, which addresses varying nutrient and electron acceptor availability throughout the biofilm structure (32), involves the intracellular tuning of c-di-GMP levels by multiple transducers that link environmental sensing to enzymatic activity (3, 33). In *Pseudomonas aeruginosa*, the redundancy of over 40 genes controlling c-di-GMP levels likely reflects the need for fine-tuned responses to a plethora of stimuli (10). However, despite the relevance of sensory domains like CHASE4, VFT, and MASE1, the protein biochemistry of nutrient sensing in *P. aeruginosa* remains poorly understood (15, 34, 35).

In the PAO1 strain, the one-component systems PA0847 and PA2072 (also named IsmP, iron-sensing membrane protein) possess a periplasmic CHASE4 sensory domain reported to bind ferric iron (15). PA0847 was previously found to regulate bacterial motility without significantly affecting biofilm formation in response to different amino acids (16). On the other hand, PA2072 responds to ferric iron (and heme) to inhibit the DGC activity of its protein partner IsmA (15).

**Fig 7:**
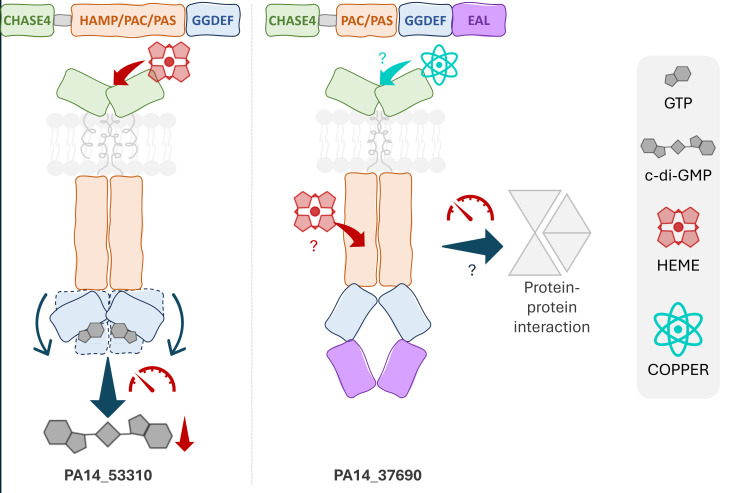
Sensing through the CHASE4 domain in the GGDEF-EAL proteins from PA14 *P. aeruginosa* characterized in this study. On the left, the domain organization of PA14_53310 (on the top) and a cartoon showing the proposed mechanism of signal transduction, where DGC activity is slowed down upon CHASE4-heme interaction. On the right, the domain organization of PA14_37690 (on the top) and a cartoon showing the possible environmental sensing activity. According to our preliminary data, it is likely that the protein interacts with heme from the cytoplasmic side and with copper from the periplasmic side, through the CHASE4 domain. Question marks in cyan-green and red are included, since more data are required to associate such *in vitro* interactions with the signal transduction. At this stage, we have no data supporting a possible role of PA14_37690 in protein-protein interaction, as reported for the PAO1 counterpart; for this, the question mark in blue is also included. The cartoon shows proteins depicted as dimers, according to literature data on this class of one-component system; the figure represents a summary of the main achievements of this work.

This study, however, reveals a fascinating versatility and specialization of these systems in the PA14 clinical strain. We found that the PA14 homolog of PA0847, PA14_53310, does not bind iron but instead binds heme via its CHASE4 domain, which in turn negatively regulates the protein’s DGC activity. On the other hand, the PA14 homolog of PA2072, PA14_37690, binds copper via its CHASE4 domain, while its interaction with heme appears to occur in the cytoplasmic portion, where the IsmP/IsmA interaction is expected (15) (a cartoon summarizing the most relevant results is reported in Fig. 7). Copper binding to CHASE PA14_37690 shows exothermic peaks whose integration gives a typical exothermic sigmoidal curve; in PA2072, ferric iron ITC shows endothermic peaks and an unexpected exothermic sigmoidal curve, thus indicating that the mechanism of binding is different (15). Copper is a Janus micronutrient, required for cell survival, attractant if needed (36, 37) but toxic or even bactericidal at high concentrations (38), probably by targeting redox homeostasis, envelope integrity, and macromolecular functionality (39). To evade copper toxicity, bacteria must sense copper and tightly regulate gene expression, biofilm, and virulence (40–43). In *P. aeruginosa*, model simulations support the presence of periplasmic copper storage to control the interplay of periplasmic and cytoplasmic pools, in response to extracellular copper stress (44). Functional studies are needed to understand whether PA14_37690 contributes to copper homeostasis involving c-di-GMP tuning.

In this study, the interaction with heme, another critical signal in the host-pathogen interaction (45), occurs in two distinct modes. In PA14_53310, heme binds to the periplasmic domain with classical globin-like coordination, suggesting a role as a redox sensor for the periplasmic heme pool, analogous to other known systems (24, 46, 47). Here, we *in vitro* show that excess heme negatively regulates the DGC activity; further studies are needed to demonstrate the out-to-in signal transduction via the CHASE periplasmic domain (Fig. 7, left panel).

In PA14_37690, the interaction with heme involves the cytoplasmic portion of the protein, which displays unusual spectroscopic features (Fig. S10), resembling those found in non-histidine five-coordinate species (23–25). The double-peaked profile reminds to the newly discovered multifactorial sensory hemoprotein NosP from *Burkholderia thailandensis*: the heme-reconstituted protein shows a UV-Vis spectrum very close to those shown in Fig. S10, with two peaks at 411 and 383 nm (48). The authors suggested that the protein populates mixed species, with that at 383 nm probably missing the conformational change required for the 411 nm coordination. Interestingly, this heme sensor has a role in biofilm regulation in this soil-dwelling bacterium, acknowledging the role of “labile heme responsive proteins” in environmental sensing (48).

In the host-pathogen interaction, heme has been classified as a “paradox”: a precious source of iron for both characters, a possible source of stress and toxicity above a threshold level (both in the environment and/or in the intracellular *milieu*) (45). *P. aeruginosa* takes advantage of extracellular heme during infections and competes for it with the host, particularly in cases of iron starvation (49, 50). For this reason, heme sensing is strategic to balance heme uptake and heme tolerance (including regulation of export, sequestration, and degradation) (45). It should be mentioned that recombinant heme proteins can be purified as “apo,” particularly heme-based sensors (22). The possibility that these proteins are heme-based gas sensors (51) cannot be ruled out; at this stage, we cannot precisely assign a role to the heme binding to PA14_53310 and PA14_37690, and therefore, further studies are needed to understand whether these proteins specifically contribute to PA14 *P. aeruginosa* heme tolerance at the infection site.

The PA14_53310 and PA14_37690 proteins appear to have different impacts on factors related to bacterial biofilm formation. Deleting the *PA14_53310* gene has phenotypes typically associated with the loss of a DGC, including reduced CR binding and increased swarming motility. Loss of PA14_37690 function has more subtle phenotypes, with reduced CR binding but no apparent impact on swarming motility. Together, these phenotypes indicate that PA14_53310 and PA14_37690 play distinct roles in the biology of this bacterium.

It is important to consider the study’s limitations. The choice of recombinant constructs and the huge allosteric control of these proteins can influence protein behavior (21, 52), and observed differences between PA14 and PAO1 homologs may partly depend on this. For instance, our PA14_53310 CHASE4 construct, designed according to (15), was insoluble (Fig. S5B), unlike its PAO1 counterpart. Furthermore, the C-terminal His-tag, while chosen to avoid N-terminal processing issues required for membrane translocation, is a potential pitfall in metal-binding studies, a limitation relevant to both this work, overcome with the validation using His-tag-free constructs, and that of (15). In conclusion, the differential ligand specificity observed in the conserved CHASE4 domains of PAO1 and PA14 strains underscores a remarkable degree of evolutionary fine-tuning. This diversification of sensory capabilities may reflect the adaptation of *P. aeruginosa* to specific niches, such as infection sites, where the ability to monitor heme availability and copper toxicity provides a strategic advantage for survival and pathogenesis.

## MATERIALS AND METHODS

### Media and reagents

Luria-Bertani (LB) medium was prepared in ultrapure water using 10 g/L NaCl (Carlo Erba), 10 g/L tryptone (BD Biosciences), and 5 g/L yeast extract (BD Biosciences). The medium was then autoclaved to ensure sterility. Solid LB medium was prepared following the same protocol by adding 15 g/L agar (BD Biosciences).

Kanamycin was prepared by solubilizing 30 mg/mL of kanamycin sulfate (Sigma) in autoclaved ultrapure water. The antibiotic was then filtered (sterile 0.22 μm filter, Biofil), aliquoted in a sterile hood, and stored at −20°C.

Chloramphenicol was prepared by solubilizing 34 mg/mL of Chloromycetin (Sigma) in ethyl alcohol (pure ≥99.8%, Sigma). The antibiotic was then filtered (sterile 0.22 μm filter, Biofil), aliquoted in a sterile hood, and stored at −20°C.

dNTPs (Thermo Fisher Scientific) 10 mM stock solution were used for plasmid mutagenesis; IPTG (isopropyl β-D-thiogalactoside; VWR) was prepared as a 1 M stock solution in autoclaved ultrapure water. The IPTG was then filtered (sterile 0.22 μm filter, Biofil), aliquoted in a sterile hood, and stored at −20°C; phenylmethylsulfonyl fluoride (Sigma) 100 mM stock solution was prepared in 99.8% 2-propanol (PanReac AppliChem) and stored at −20°C; NiSO_4_ (Sigma) 0.1 M stock solution was used to equilibrate the Ni^2+^-IMAC column and was prepared in ultrapure water and stored at room temperature; DDM (n-dodecyl-β-D-maltoside, Avanti Research) 5% stock solution was prepared in ultrapure water and stored at 4°C.

MgCl_2_ (Carlo Erba), MnCl_2_ (Sigma), CuCO_4_ (Sigma), and FeCl_3_ (Sigma) 100 mM stock solutions were prepared in ultrapure water and stored at room temperature, protected from light sources with silver paper.

GTP was prepared according to reference 53.

Hemin chloride (Sigma) was prepared by dissolving ~10 mg of powder in 4 mL of 10 mM NaOH; after vortexing and 10 min of centrifugation at 12,000 rpm, the suspension was filtered (sterile 0.22 μm filter, Biofil). To determine the concentration of the solution, 3 μL of the hemin solution is diluted to 800 μL with 100 mM Tris, pH 9.0, and the UV-Vis spectrum was collected in a 1 cm path quartz cuvette (Hellma); absorbance at 390 nm was used, considering a molar extinction coefficient of ɛ_390_ = 50 M^−1^ cm^−1^.

### Plasmids and strains

DNA manipulation (plasmid amplification and mutagenesis) has been carried out with *Escherichia coli* NEB 5-alpha Competent *E. coli* (New England Biolabs) cells. Expression constructs are summarized in Fig. 1, and plasmid details are reported in Table S2. The QuikChange Lightning Site-Directed Mutagenesis Kit (Agilent Technologies) was used for plasmid mutagenesis.

Protein expression has been done with *E. coli* BL21 (DE3) and BL21 (DE3) pLysS strains, as indicated.

### Protein expression and purification

Expression strains have been transformed with the corresponding expression vector, and protein expression has been carried out in LB medium starting from a single colony. Colony inoculation was carried out overnight at 37°C at 180 rpm; the inoculum was diluted 1:100 and the growth was carried out at 37°C until OD_600_ = 0.8, then 1 mM IPTG was added to induce gene expression and the temperature was moved to 22°C. In Table S2, the strains, the antibiotics, and the time of induction used for each construct are reported. Cells were then harvested by centrifugation at 5,000 rpm for 20 min and stored at −20°C.

Each pellet was resuspended in the corresponding lysis buffer (40 mL/L of culture, see details in Table S3) and lysed by sonication on ice. For membrane proteins, cell lysate was centrifuged 5 min at 5,000 rpm in order to remove any cellular debris. The supernatant was then ultracentrifuged at 35,000 rpm (~125 × 10^3^ × *g*) for 1 h. The obtained pellet was suspended in buffer 50 mM Hepes, pH 7.6, 300 mM NaCl, 2.5% glycerol, 1% DDM (Avanti Research) using a homogenizer. The protein was then stirred at 4°C for 2 h to promote detergent-mediated solubilization and was then diluted 1:1 with buffer 50 mM Hepes, pH 7.6, 300 mM NaCl, 2.5% glycerol, and ultracentrifuged for 1 h at 35,000 rpm; the obtained supernatant was kept at 4°C overnight. For water-soluble constructs, pellets were suspended in the corresponding lysis buffer, lysed by sonication on ice, and centrifuged 40 min at 12,000 rpm.

Each supernatant was purified by affinity chromatography using a HisTrap column (Cytiva) loaded with Ni^2+^ (hereinafter Ni^2+^-IMAC) and equilibrated with 50 mM Hepes, pH 7.6, 300 mM NaCl, 2.5% glycerol, and 0.01% DDM. Elution was carried out by increasing the imidazole concentration, as detailed in Table S3. Fractions containing the protein were analyzed through SDS-PAGE and collected. Imidazole was removed with PD MiniTrap G-25 (Cytiva) (hereinafter desalting column) and then the proteins were loaded onto a Superdex 200 Increase 10/300 GL (Cytiva), if indicated. Both the desalting and Superdex columns have been equilibrated with the corresponding final buffer reported in Table S3.

All the purified proteins were flash-frozen in liquid nitrogen and stored at −20°C. Protein concentration has been determined using the extinction coefficients in Table S3.

### Continuous kinetic assays using CD

DGC activity of PA14_53310 was analyzed by real-time kinetics using CD, as previously published (20), using a JASCO J-815 spectropolarimeter. The assays were carried out starting from 450 μL of kinetic buffer (50 mM Hepes, pH 7.6, 300 mM NaCl, 2.5% glycerol, 0.01% DDM, 2 mM MgCl_2_, 2 mM MnCl_2_) containing 2 μM of the DDM-solubilized protein, with or without 10 μM hemin chloride (Sigma), in a 1 cm path quartz cuvette (Hellma). Before adding the substrate into the cuvette, a spectrum was recorded using the following parameters: 240 nm–340 nm range, 100 mdeg sensitivity, data pitch 1 nm, scanning speed 100 nm/min, response 4 s, band width 5.0 nm. The instrument was then set at 285 nm, and the time-course acquisition mode was started (data pitch: 10 s); after 60 s from the start of recording the c-di-GMP signal in a real-time fashion, 50 μL of a solution containing GTP (100 μM in the final volume of 500 μL) was added to the cuvette, continuing to record the signal for 2 h. The v_0_ was calculated by linear fit according to reference 54. The aforementioned parameters were also used to collect the CD spectra of standard solutions of c-di-GMP to derive the calibration curve used to convert the CD_285_ signal into [c-di-GMP] µM. Experiments were done in duplicate.

### Discontinuous kinetic assays using reverse-phase high-performance liquid chromatography (RP-HPLC)

Catalytic activity of PA14_53310 and PA14_37690 were evaluated by RP-HPLC. Two micromolar of the DDM-solubilized protein was incubated 20 min at 25°C in the kinetic buffer (50 mM Hepes, pH 7.6, 300 mM NaCl, 2.5% glycerol, 0.01% DDM, 2 mM MgCl_2_, and 2 mM MnCl_2_) with or without 10 μM of hemin chloride (Sigma). After the incubation, 100 μM of GTP (GE Healthcare) was added to the mixture to probe DGC activity, or 30 µM c-di-GMP (or a mixture of both nucleotides) to probe the PDE activity (for the sole PA14_37690, which has the EAL domain downstream of the GGDEF domain).

Kinetic assays were stopped at different time points by adding EDTA (1:1, 50 mM, pH 6.0) and boiling at 95°C for 10 min. Samples were centrifuged for 10 min at 13,000 rpm, and the protein precipitate was removed with 0.2 μm filters (Millex-LG 13 mm, Merck). The reaction products were separated using a 150 × 4.6 mm reverse-phase column (Prevail C8, Grace Davison Discovery Science, particle size 5 μm) equilibrated in 100 mM phosphate buffer pH 5.8/methanol (98/2, vol/vol, 1 mL/min) as the mobile phase, and the UV detector was set at 254 nm.

Standard solutions of each nucleotide were prepared and run at the beginning of each data collection. Experiments were done in duplicate.

### UV-Vis spectroscopy of heme binding

*In vitro* heme binding was assayed using protein solutions in the final buffer reported in Table S3, at the following concentrations: for PA14_53310, 2.1 μM and 4.6 μM for the full-length and the CHASE4, respectively; for PA14_37690, 4.5 μM for the full-length and 3.6 µM or 10 μM for the cytoplasmic and CHASE4 constructs, respectively.

Five hundred microliters of each protein solution and 500 μL of the corresponding buffer were “titrated” with increasing concentrations of a freshly prepared solution of hemin chloride (Sigma, see media paragraph for preparation protocol) in 10 mM NaOH. Titrations were performed in a 1 cm path quartz cuvette (Hellma) by using a Varian Cary 50 UV instrument, and each spectrum was recorded between 250 and 800 nm. Experiments were done in duplicate.

### ITC assays

ITC assays were performed using a Microcal Peaq-ITC (Malvern) as follows: the cell was loaded with 200 µL of 6 µM protein, and the injection syringe with 40 µL of 100 µM of CuSO_4_ or 250 µM of FeCl_3_, if not otherwise indicated. Metal salts were resuspended in the same final buffer used to store the purified protein. Proteins were dialyzed against the purification buffer for 18 h (2 × 500 mL of buffer exchange). The injection volume was 2 µL (with the exception of the first, 0.4 µL), the stirring speed of 750 rpm, and the spacing was 150 s between each injection. Experiments were done in triplicate.

In case of binding, data were fitted with a one-site binding mode equation, using Origin-MicroCal Software.

### DSF assays

DSF assay has been performed using Sypro Orange dye (2.5×, Thermo Scientific). The fluorescent signal is measured using a Real-Time PCR machine (CFX Connect Real-Time PCR system, Bio-Rad). Protein and metals were mixed to reach a final volume of 100 µL per sample. The PA14_37690 CHASE4 has been used at a concentration of 4 µM, while every salt at 70 µM. In addition, SYPRO has been diluted 1:10 and, from this dilution, 0.5 µL was added to each sample. The starting temperature was programmed at 25°C to reach the final temperature of 95°C with 0.4°C increments every 30 s (excitation 450 nm–490 nm; detection 560 nm–580 nm). All samples were run in triplicate. Curve fitting has been performed using the Boltzmann sigmoid function.

### Thermal melting experiments

Thermal melting assays of PA14_37690 were performed using a JASCO J-815 spectropolarimeter. Samples were prepared in a final volume of 250 µL and loaded into a sealed 0.1 cm path quartz cuvette (Hellma), with the protein at a concentration of 1.5 µM, while the ligand (CuSO_4_) was 70 µM. The melting temperature of the protein was measured by following the signal at 222 nm in the temperature range 25°C–90°C.

### Homology modeling

Protein sequences for PA14_37690 and PA14_53310 were retrieved from UniProt and analyzed for domain content. Transmembrane helices were predicted using the TMHMM algorithm (55). Domain boundaries were assigned by scanning each sequence against multiple databases: Pfam (pfam.xfam.org [56], InterPro [57], and the NCBI Conserved Domain Database (CDD) via CD-Search [58]). The Pfam PF05228 profile for CHASE4 was used to identify CHASE4 domains, while InterProScan and CDD provided complementary annotations.

Structural modeling of PA14_53310 was performed using AlphaFold2 (AF2) (59) with default parameters. The full-length PA14_53310 sequence was input to AF2 to generate an initial 3D model, which was evaluated for confidence (pLDDT) scores and global fold plausibility. The resulting model was then imported into PyMOL and refined using the MODELLER program (60) via the PyMod3 interface (61). In PyMod3, comparative modeling was applied by aligning the AF2 PA14_53310 model to the PA2072 CHASE4 structure to generate homology models using MODELLER’s loop refinement routines. Several MODELLER-generated models were assessed by discrete optimized protein energy scores, and the lowest-energy model was selected. MODELLER refinement via PyMod3 further improved local geometry (especially loops and side chains) without altering the overall fold or boundary positions.

Structural visualization and comparison were performed in PyMOL and UCSF ChimeraX. The PA2072 CHASE4 crystal structure (PDB: 8Z84) was used as a reference for fold and boundary confirmation. The AlphaFold2/Modeller model of PA14_53310 was superposed onto 8Z84 to compare domain architectures. Root-mean-square deviation (RMSD) and TM-align (62) were used to quantify structural similarity. All figures were prepared by illustrating membrane helices (from TMHMM) as ribbons and domains as colored boxes to emphasize topology and boundaries.

### Congo red assay

The Congo red dye uptake assay was adapted from a previously published protocol (62). Briefly, M8 agar (1%) plates supplemented with CR solution (final concentration CR at 0.04 mg/mL with 0.01 mg/mL Coomassie blue) were spotted with 2 µL of an overnight culture and incubated at 37°C for 16 h. Plates were then grown at room temperature for an additional 48–72 h, then photographed.

### Cyclic-di-GMP quantification

c-di-GMP levels were quantified via liquid chromatography-mass spectrometry at the Michigan State University Mass Spectrometry and Metabolomics Core, as reported (26). Briefly, cells were harvested from swarm plates after 16 h of growth and c-di-GMP levels were normalized to the dry weight of cell pellets after nucleotide extraction. All experiments were performed in triplicate with three technical replicates per strain.

### Swarm assay

Swarming assays were performed as reported (63). Briefly, M8 medium was supplemented with 0.5% agar. Swarm plates were inoculated with 2 µL of an overnight culture and incubated at 37°C for 16 h, then photographed and quantified as reported (63).

## SUPPLEMENTAL MATERIAL

Supplemental materials jb.00370-25-s0001.pdf Figures S1 to S13, Table S1, and Supplemental methods.

ASM does not own the copyrights to Supplemental Material that may be linked to, or accessed through, an article. The authors have granted ASM a non-exclusive, world-wide license to publish the Supplemental Material files. Please contact the corresponding author directly for reuse.
