# Supplementary material for: The versatility of the CHASE domain in one-component systems of c-di-GMP signal transduction from P. aeruginosa
Source: J Bacteriol. 2026 May 28;208(6):e00370-25. doi: 10.1128/jb.00370-25 (PMC13277305; doi:10.1128/jb.00370-25)
Supplement: Supplemental materials — Figures S1 to S13, Table S1, and Supplemental methods. [file jb.00370-25-s0001.pdf]

SUPPLEMENTARY MATERIALS

The versatility of the CHASE domain in one-component systems of c-di-GMP signal transduction from *P. aeruginosa*.

Simone Angeli[1*], Chiara Scribani-Rossi[1*], Giulio Colasanto[1], Sharon Spizzichino[1], Serena Rosignoli[2], Alessandra Giorgi[1], Francesca Cutruzzolà[1], Alessio Paone[1], Angela Tramonti[3], Roberto Contestabile[1], Robert Montoya[4], George A O'Toole[4], Alessandro Paiardini[1], Serena Rinaldo[1#]

[1]Department of Biochemical Sciences A. Rossi Fanelli, Sapienza University of Rome, Italy

[2]Centre for Regenerative Medicine "Stefano Ferrari", Department of Life Sciences, University of Modena and Reggio Emilia, 41125 Modena, Italy

[3]Istituto di Biologia e Patologia Molecolari, Consiglio Nazionale delle Ricerche, Rome, Italy

[4]Geisel School of Medicine at Dartmouth, Hanover, New Hampshire 03755, USA

Corresponding author: serena.rinaldo@uniroma1.it

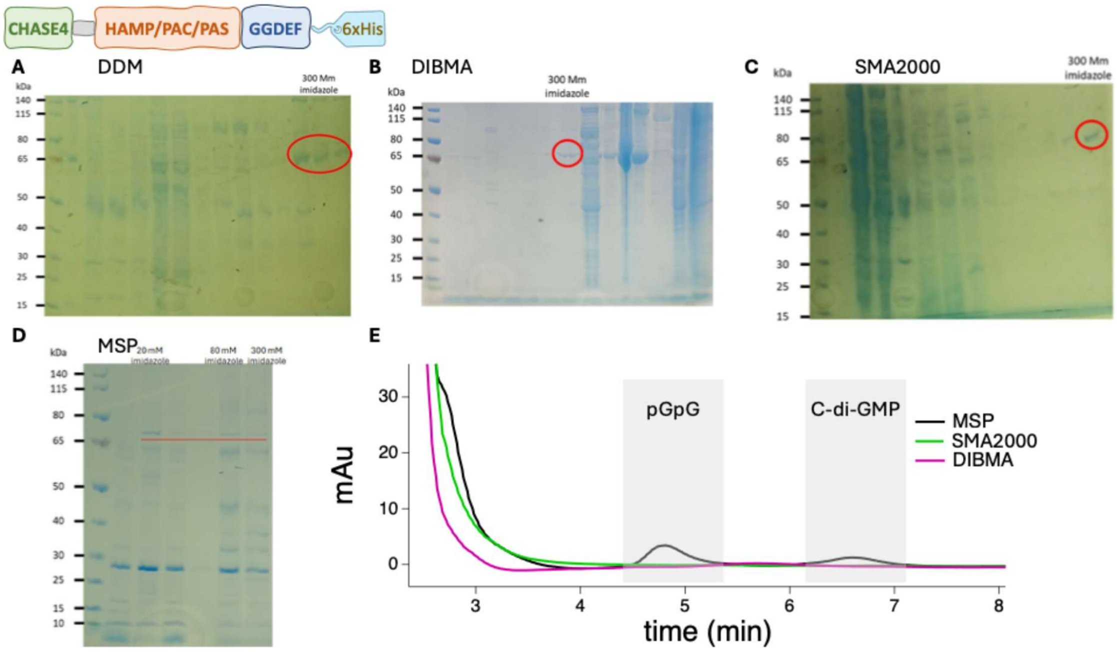

Figure S1. Isolation and kinetics of PA14_53310 nanodisc. Purification of PA14_53310 extracted with DDM detergent (A), or assembled in DIBMA (B), or SMA2000 (C) or MSP (D) nanodisc after detergent extraction. Some protein degradation was observed upon MSP nanodisc assembly; the band around 24kDa is the MSP1E3D1 protein. Each nanodisc preparation was assayed for DGC activity (E). The little activity observed in the MSP sample (black line) was obtained by assembling MSP-PA14_53310 nanodiscs under DGC turnover conditions (i.e. in the presence of divalent metals and excess GTP), where dimerization is expected to occur to enter catalysis. After dialysis and isolation of the nanodisc, kinetics have been carried out in the presence of the PDE RocR, which degrades c-di-GMP into pGpG, to minimize possible product inhibition of DGC catalysis. This set up led to isolating a small fraction of active protein, too low for further biochemical characterization.

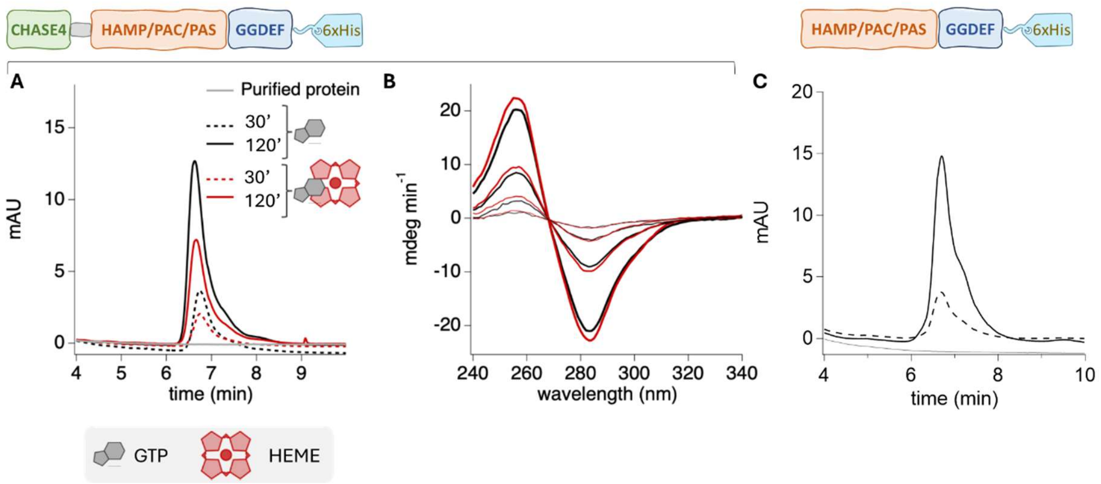

Figure S2. Effect of heme on DGC activity of PA14_53310. (A) DGC assay with 2 μM PA14_53310 solution incubated with manganese and 100 μM GTP for 30 and 120 minutes (dashed and continuous black lines, respectively); c-di-GMP content was evaluated by RP-HPLC. The same assay was repeated in the presence of 10 μM heme solution (dashed and continuous red lines). (B) CD spectra of c-di-GMP standard solutions (5, 10, 20, 40 μM) with or without excess heme (red and black lines, respectively). (C) DGC assay with 2 μM cytoplasmic PA14_53310 solution incubated with manganese and 100 μM GTP for 30 and 120 minutes (dashed and continuous black lines, respectively); c-di-GMP content was evaluated by RP-HPLC.

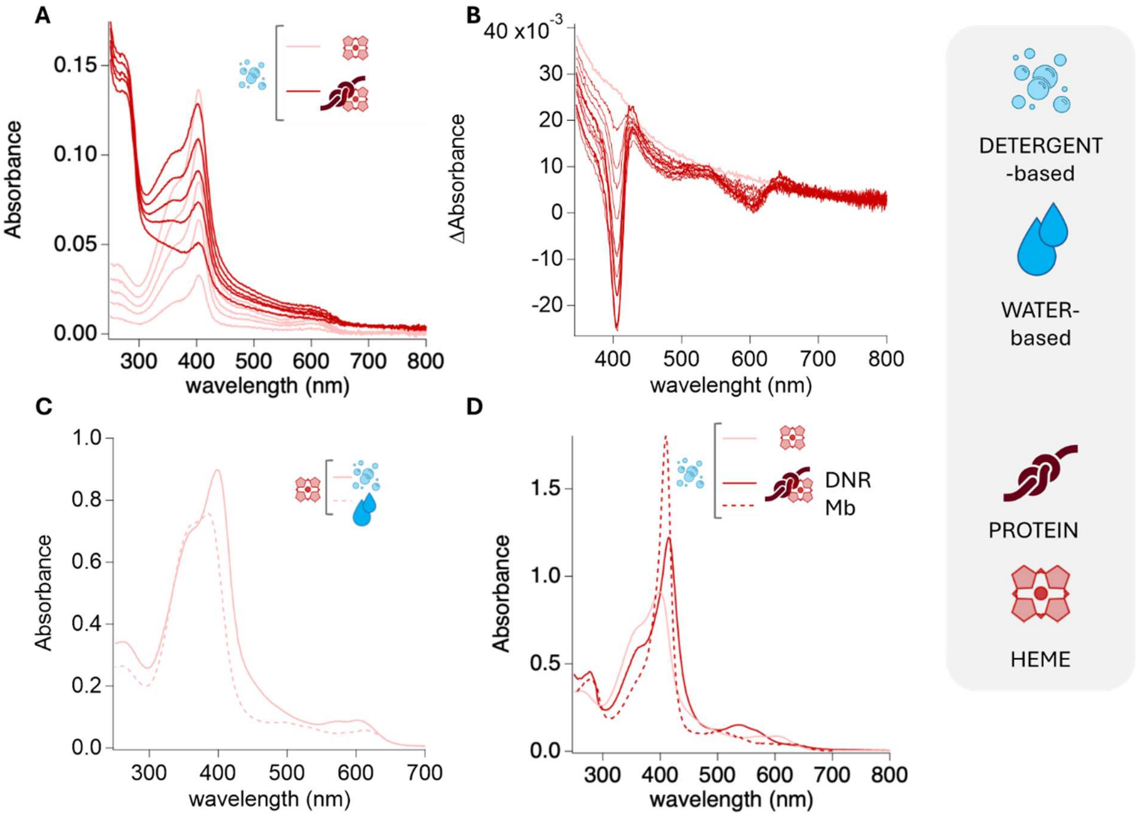

Figure S3. UV-Vis spectra of hemeproteins. (A) Heme titration of PA14_53310. In the Figure UV-Vis spectra of 2.1 μM PA14_53310 upon addition of increasing amount of hemin solution (red lines); for comparison, UV-Vis spectra of the same amount of hemin are reported (pink lines). (B) Differential spectra of the species reported in panel A. A negative peak above 400 nm could be representative of protein-dependent heme iron coordination. (C) UV-Vis spectra of 10 μM Venus Fly Trap domain of RmcA from *P. aeruginosa* (Paiardini et al., 2018) in the presence of 10 μM hemin (red lines), collected in a water-based or detergent-based buffer (dashed or continuous lines, respectively). This protein does not bind heme and it has been used as negative control. (D) UV-Vis spectra of 10.7 μM DNR from *P. aeruginosa* (Giardina et al., 2011) with 1 equivalent of hemin (red continuous line), 10 μM Horse Myoglobin (Sigma), 10.7 μM hemin, all in detergent-based buffer. While hemin shows a peak at 399 nm, not found in the water-based spectrum, DNR and Myoglobin spectra superpose with those obtained in water-based buffer, as previously published.

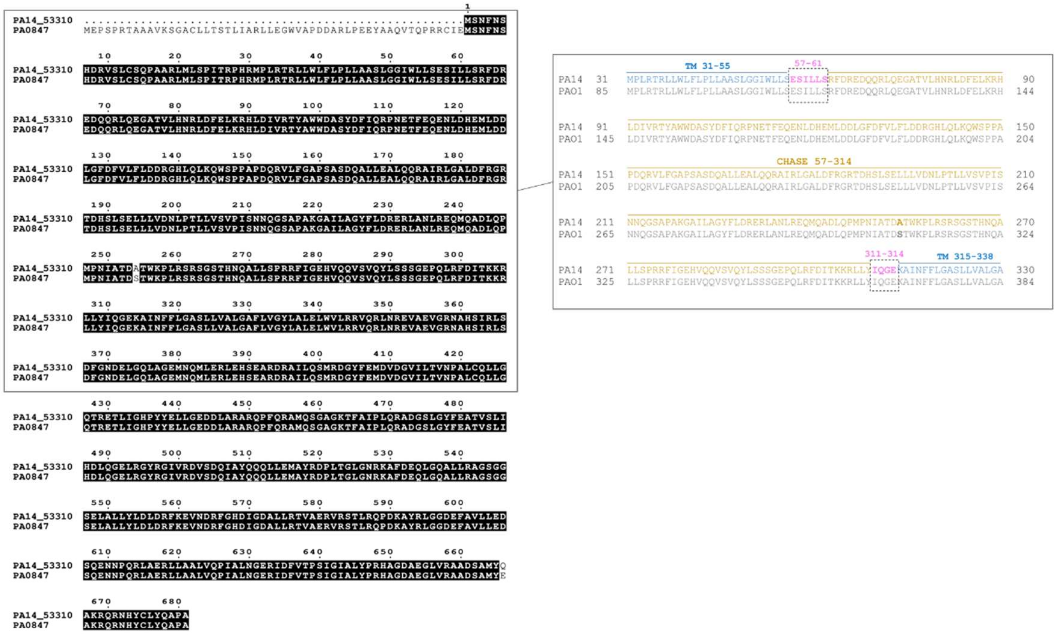

Figure S4. Sequence alignment of PA14_53310. Sequence of PA14_53310 gene was aligned with the PAO1 counterpart i.e. PA0847, previously characterized. PA0847 bears 54 residues extra sequence in the N-terminal; PA14_53310 is predicted to miss the signal peptide required for membrane translocation, as also confirmed by mass spectrometry of the SDS-PAGE band (data not shown). In the blow-up the transmembrane (TM, in blue) and CHASE4 domains are highlighted; in light brown the residues selected to isolate the PA14_53310 CHASE4 construct. In magenta the extra residues aligning with the interval used to isolate the CHASE4 domain in PA0847 (Zhan et al., 2024).

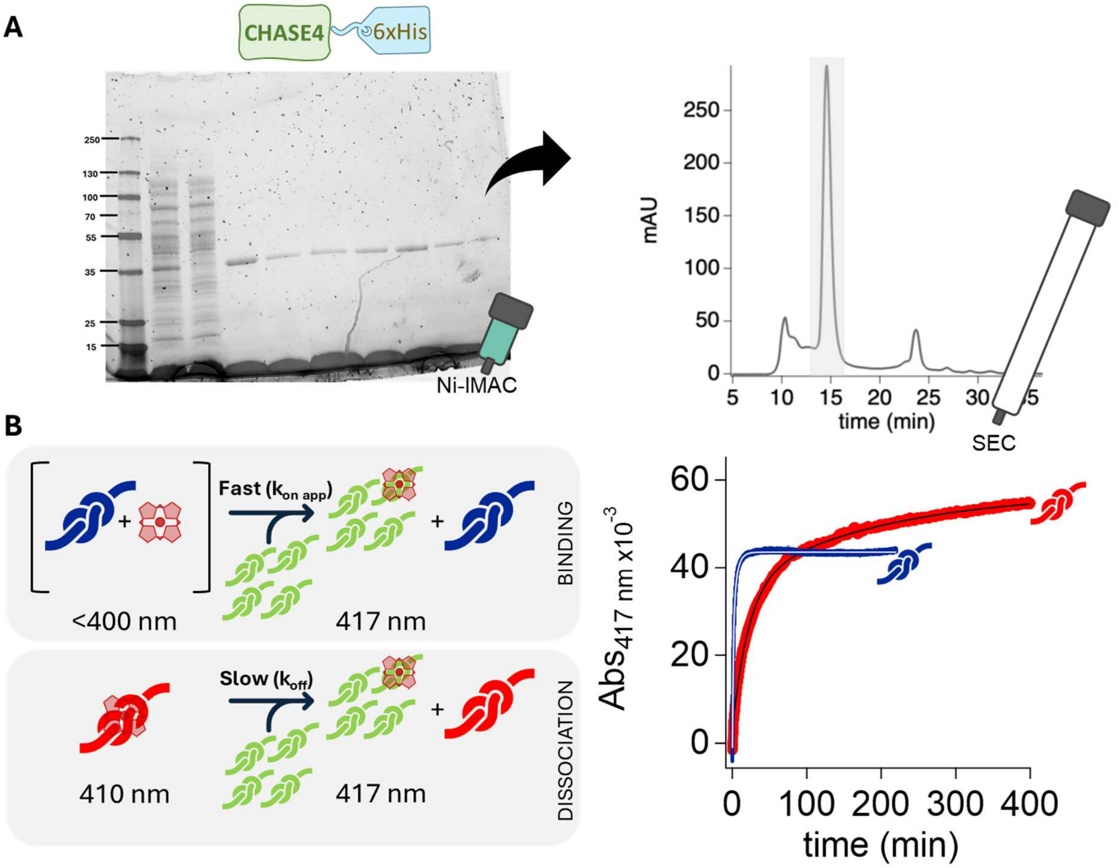

Figure S5. Purification of CHASE4 domain from PA14_53310. (A) A construct encompassing residues from 57 to 314 of PA14_53310 gene was expressed as (C-terminal) His tagged protein. Purification was carried out by Ni^{2+}-IMAC (on the left the SDS-PAGE of eluted fractions), followed by SEC separation (on the right, highlighted in grey the peak collected and used for this study). (B) Heme dissociation from 1 µM ferric PA14_53310 CHASE4 heme complex, obtained after incubation of the protein with 1 equivalent of heme for 1h at 16 °C; in red, the timecourse of heme transfer to 5 molar excesses of apo DNR from *P. aeruginosa*, “trapping” the dissociated heme. The kinetics was followed at 417 nm, where the maximal absorbance change is expected (Giardina et al., 2008); rates have been calculated by fitting the data (open circle) with a two-exponential equation (bold continuous line), yielding k_{off1}=1.5±0.6x10^{-5} s^{-1} and k_{off2}=5.5±2.1x10^{-4} s^{-1}. In case of heme dissociation from hemeprotein, as in the red trace, the process is expected to be slow, being rate-limited by the k_{off} rate of heme from the source protein. As previously observed for other hemeproteins, the biphasicity is likely due to the intrinsic heme rotational disorder (La Mar et al., 1978),

As control, the experiment has been repeated with the RmcA-VFT (His-tagged) construct incubated with 1 equivalent of heme (in blue in the Figure). In this case, where a mixed solution of protein+heme is present, the observed kinetics is, as expected, relatively fast, yielding apparent $k_{on\ app}$ ~ 10^{-2}-10^{-3} s^{-1}. The rate of ferric heme binding to a hemeprotein is “apparent”, being likely rate-limited by the heme-heme dissociation (aqueous solution of heme populates stacked complexes rather than monomeric entities, Golnak et al., 2015).

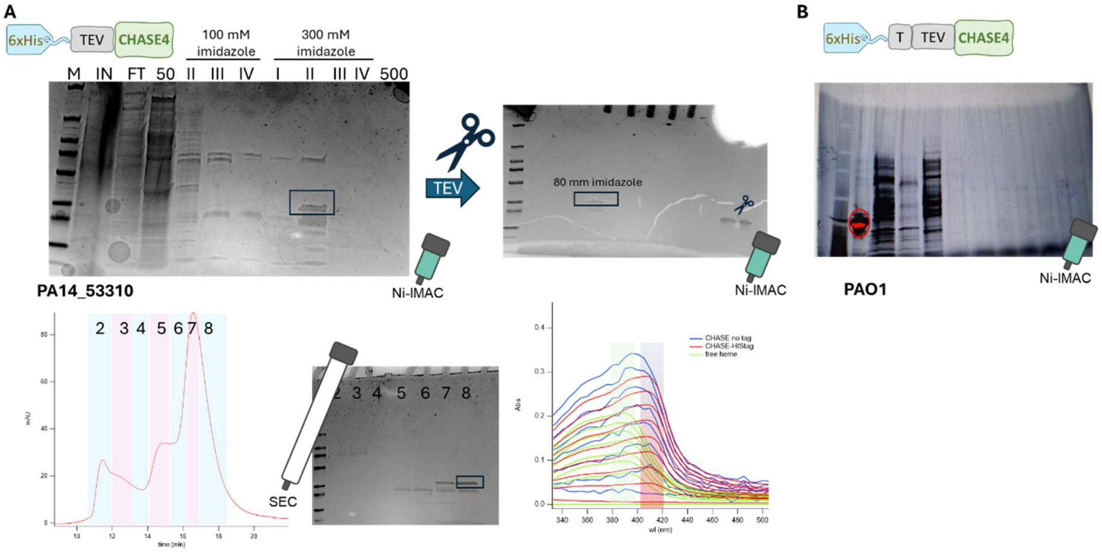

Figure S6. N-terminal tagged variants of the CHASE4 domain from PA14_53310 gene. (A) The construct purified and characterized in Figure S5 was expressed as a (N-terminal) His tagged protein, including the consensus for the TEV protease between the His-tag and the CHASE4 ORF. As shown on the top left, the protein is highly unstable and prone to proteolysis. The His-tag was successfully removed (top on the left) but the yield and the quality of the sample was very poor. To remove any trace of excess imidazole (which may interfere with the subsequent heme titration) and improve the purity of the sample, the protein was further SEC separated on Superdex75 (left, bottom). Fraction 8 was then used for heme titration (blue traces in the panel on the right, bottom). For comparison, spectra of free heme and of the His-tagged variant with increasing amount of heme have been superposed, after proper normalization (green and red traces, respectively). Both proteins peak differently and above 400 nm as compared to free heme.

(B) A construct encompassing residues from 61 to 311 of PA14_53310 gene was expressed as (N-terminal) His tagged protein, thus recapitulating the strategy used for PA0847 counterpart (corresponding to residues 117-364, as previously published in Zhan et al., 2024). Unfortunately, the protein was insoluble and the attempts to extract it from the inclusion bodies were unsuccessful.

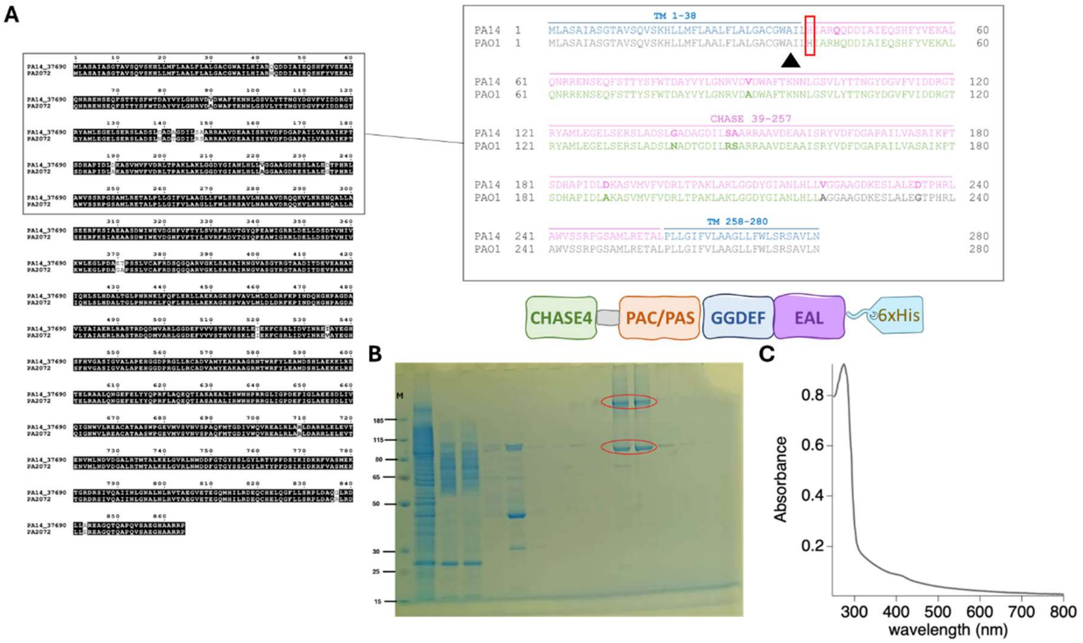

Figure S7. Purification of PA14_37690. (A) Sequence of PA14_37690 gene was aligned with the PAO1 counterpart i.e. PA2072 (also named IsmP), previously characterized (Zhan et al., 2024). In the blow-up the transmembrane (TM, in blue) and CHASE4 domains are highlighted. PA14_37690 is predicted to have a 36 residues signal peptide required for membrane translocation (black triangle); its cleavage has been confirmed by mass spectrometry of the SDS-PAGE band. It should be mentioned that this technique is based in tryptic digest and the first peptide recovered is downstream Arginine 42, suggesting that in the sample no cleavable peptide was available upstream this residue (data not shown). In magenta and in green the region of the sequence used to isolate the CHASE4 domain in PA14_37690 (this work) and PA2072 (Zhan et al., 2024), respectively. In red, Histidine 39 is boxed. Residues substitutions are in bold.

(B) Full-length protein was detergent-extracted and purified by Ni^{2+}-IMAC. SDS-PAGE of eluted fraction is shown, with the 300 mM imidazole pool used in this work (after buffer exchange with desalting column). Both bands (red circles) have been verified by mass spectrometry. (C) UV-Vis spectrum of purified PA14_37690. The small shoulder above 400 nm suggests the presence of a cofactor/ligand.

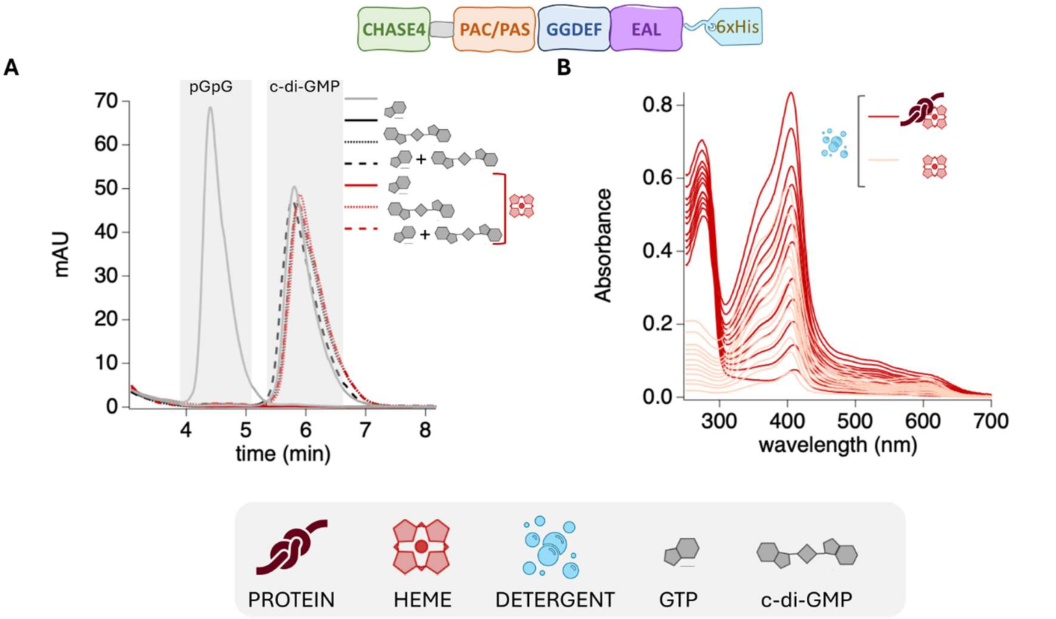

Figure S8. Characterization of PA14_37690. (A) Enzymatic assay carried out with 2 μM PA14_37690 solution incubated with manganese and 100 μM GTP, or 30 μM c-di-GMP, or 30 μM c-di-GMP+100 μM GTP 120 minutes (continuous, dotted and dashed lines, respectively); the nucleotide content of each reaction has been separated by RP-HPLC and the corresponding chromatogram is depicted; chromatograms of standard solution of c-di-GMP and pGpG are also present (grey lines). The same assay was repeated in the presence of 50 μM heme solution (red lines). No catalytic activity was observed. (B) UV-Vis spectra (upper panel) of 4.5 μM PA14_37690 in the presence of increasing amount of hemin; for comparison the corresponding hemin spectra are also included (pink lines).

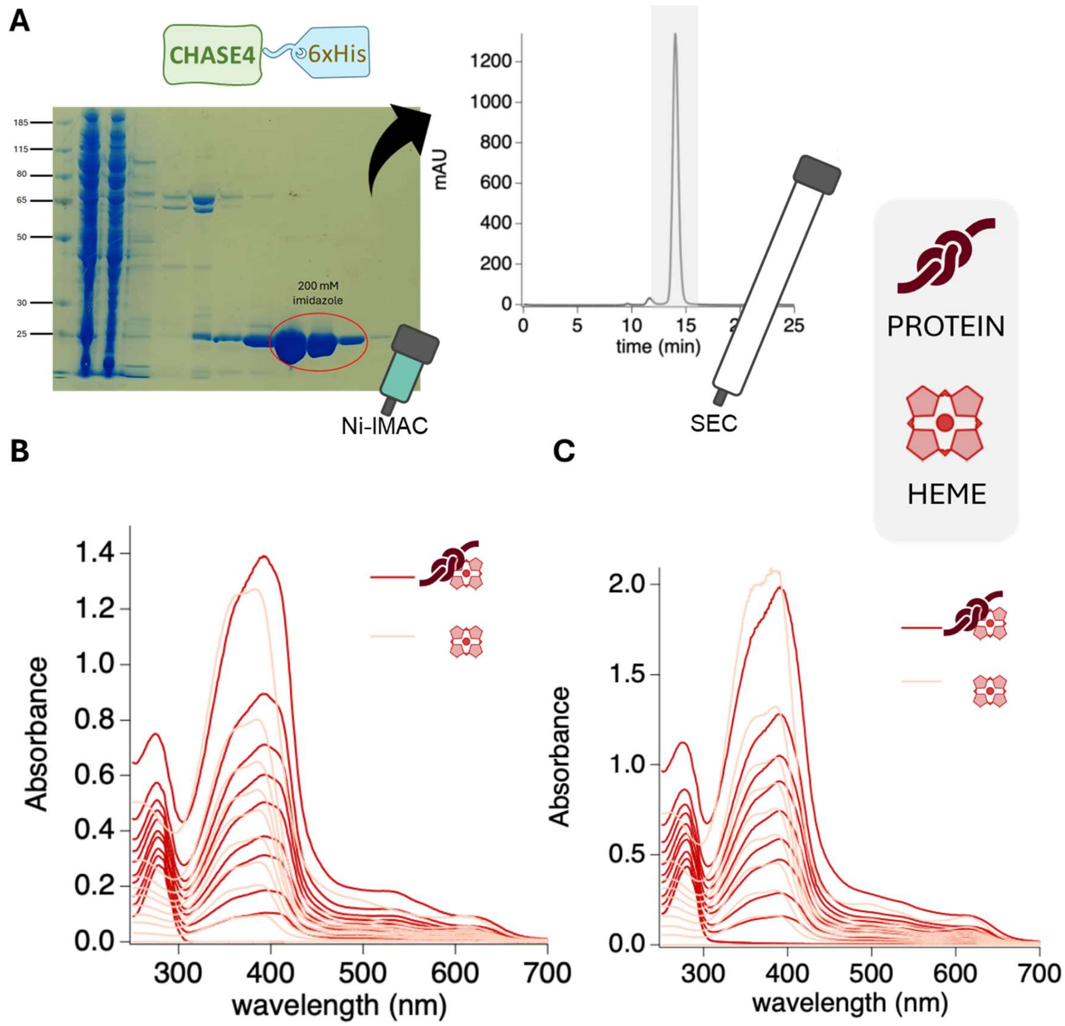

Figure S9. Purification and heme binding of PA14_37690 CHASE4 domain. (A) To isolate the CHASE4 domain, a construct encompassing residues from 39 to 257 of PA14_37690 gene was expressed as (C-terminal) His tagged protein. Purification was carried out by Ni^{2+}-IMAC (on the left the SDS-PAGE of eluted fractions), followed by SEC separation (on the right, highlighted in grey the peak collected and used for this study). (B) UV-Vis spectra of 10 µM CHASE4 protein described in (A) in the presence of increasing amount of hemin solution (red traces); the spectra of the corresponding hemin solution with the sole buffer are also included in pink. (C) UV-Vis spectra of the heme titration of the Venus Fly Trap domain described in Figure S3C, carried out in a water-based buffer.

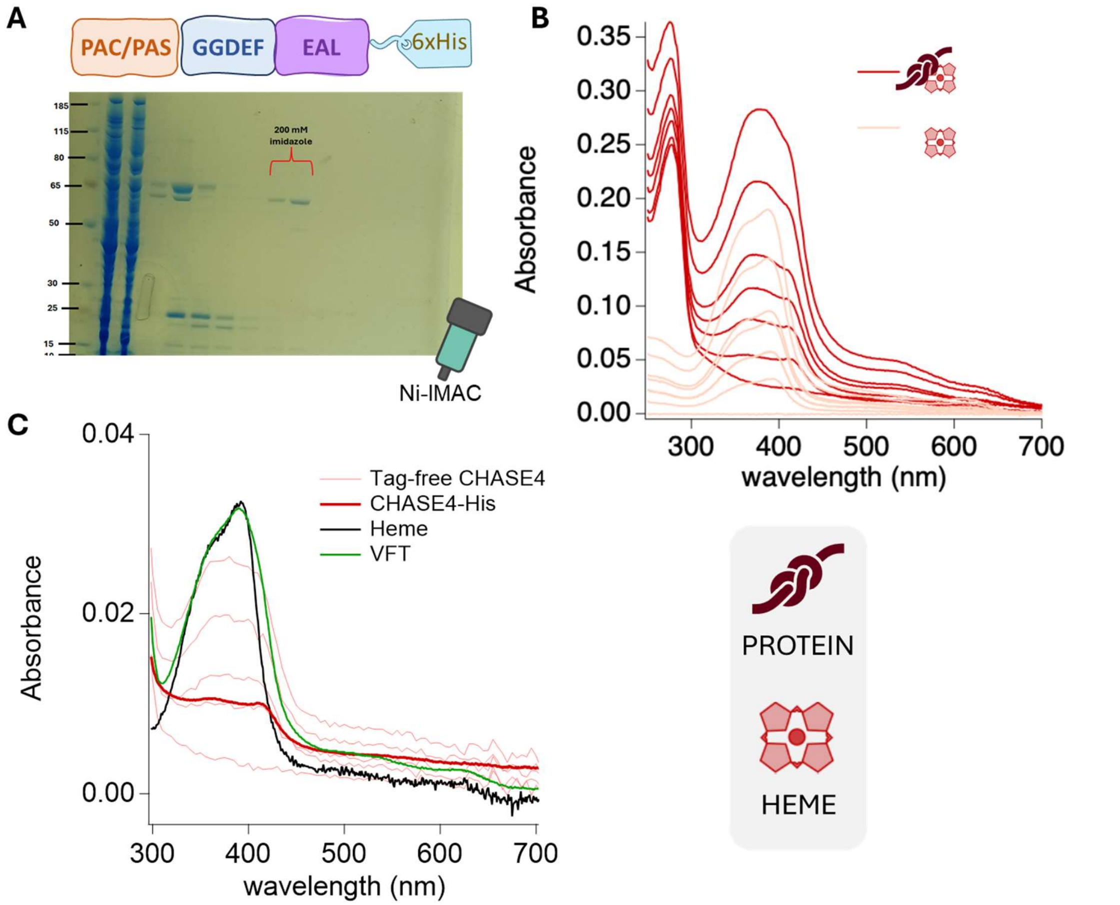

Figure S10. Purification and heme binding of PA14_37690 cytoplasmic portion. (A) To isolate the cytoplasmic portion, a construct encompassing residues from 274 to 864 of PA14_37690 gene was expressed as (C-terminal) His tagged protein. Purification was carried out by Ni^{2+}-IMAC (on the left the SDS-PAGE of eluted fractions); the low yield hampered a subsequent SEC separation and imidazole was removed by desalting column. (B) UV-Vis spectra of 3,6 μM of the cytoplasmic portion of PA14_37690 described in (A) in the presence of increasing amount of hemin solution (red traces); the spectra of the corresponding hemin solution with the sole buffer are also included in pink. (C) A construct including the ORF described in (A) but with a cleavable N-terminal His-tag was purified and the tag removed (see Figure S13); despite the low yield, heme titration was carried out; the corresponding spectra (dark pink traces in Figure) resemble those reported in panel B (one sample spectrum was included in panel C, for rapid comparison; red trace).

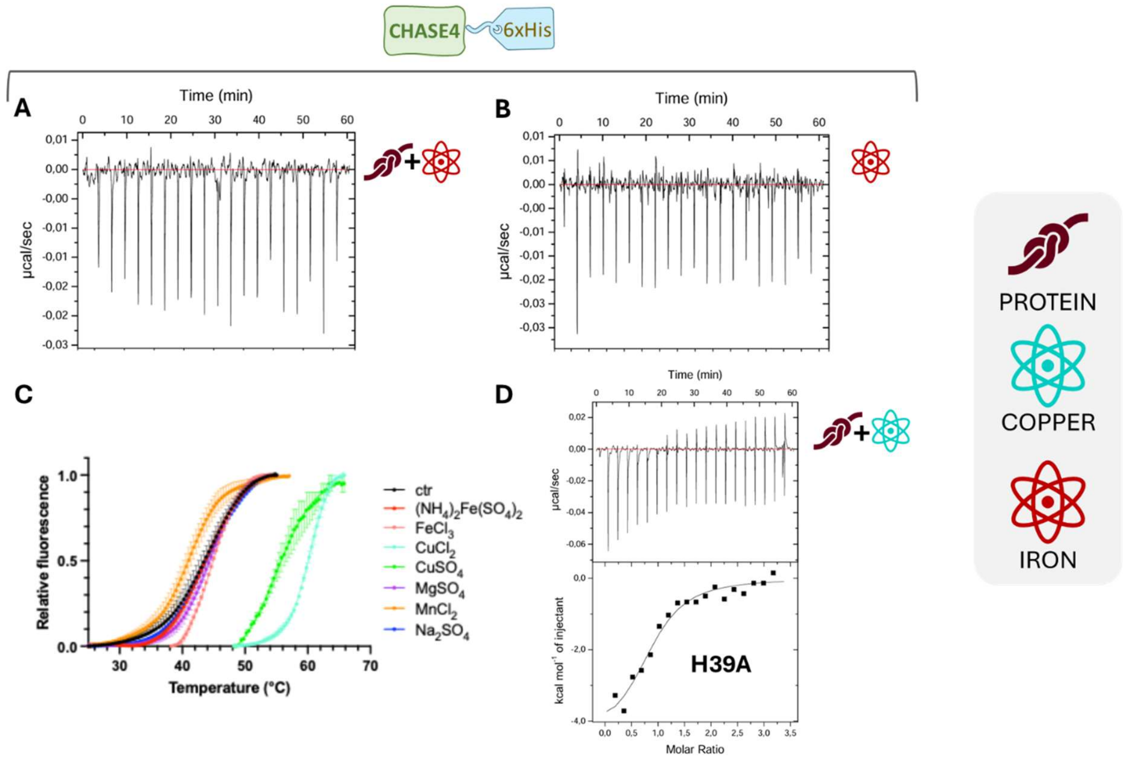

Figure S11. Interaction of the CHASE4 domain from PA14_37690 with metals. (A) ITC experiment carried out by titrating 15 µM CHASE4 from PA14_37690 with $FeCl_3$ indicates that there is no binding. (B) ITC experiment showing the injection of $FeCl_3$ into the sole buffer, used as control. (C) Effect of different metals on the thermal stability of CHASE4 from PA14_37690, assayed by DSF. The curves shown in the figure are the average with standard error bars of three independent experiments, each performed in triplicate. Data were fitted to the Boltzmann equation to obtain the melting temperatures, represented in Figure 5B. (D) ITC experiment carried out by titrating the H39A variant of the CHASE4 from PA14_37690 with $CuSO_4$ showing a one-site binding.

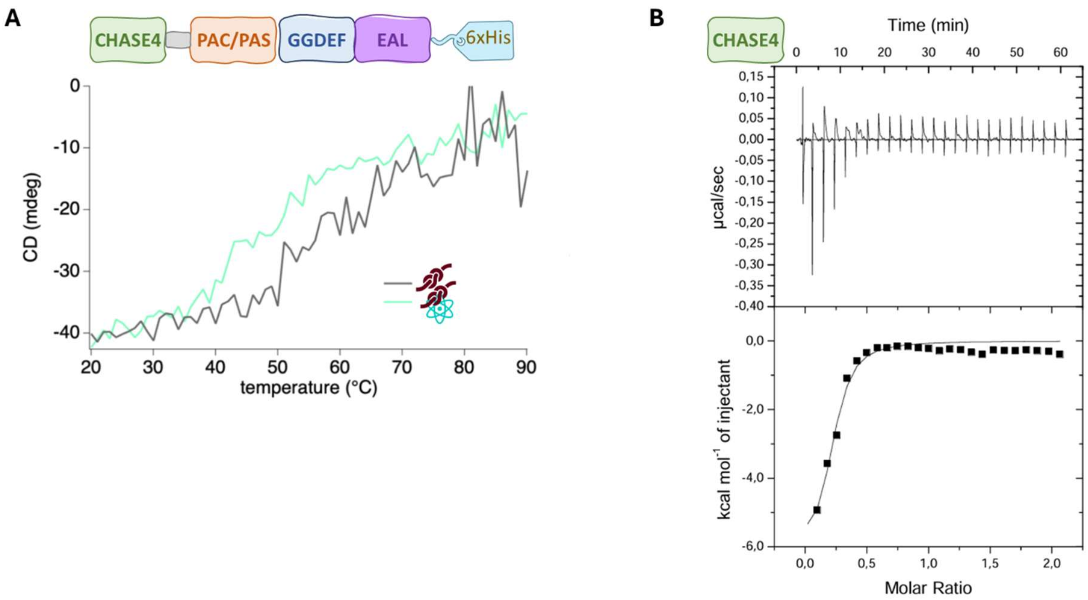

Figure S12. Interactions of PA14_37690 variants with copper. (A) Thermal melting assayed by CD spectroscopy at 222 nm, showing the denaturation of the full-length PA14_37690 with or without excess $CuSO_4$ (green and black traces, respectively). (B) ITC experiment carried out by titrating the His-tag free variant of the CHASE4 from PA14_37690 with $CuSO_4$, fitted with the one-site binding equation (yielding a K_D=1.17±0.46 μM and a stoichiometry of 0.32±0.11). The reduced stoichiometry could be ascribed to a partial removal of metal bound in the as-purified protein upon dialysis, suggesting heterogeneity of the metal binding site(s).

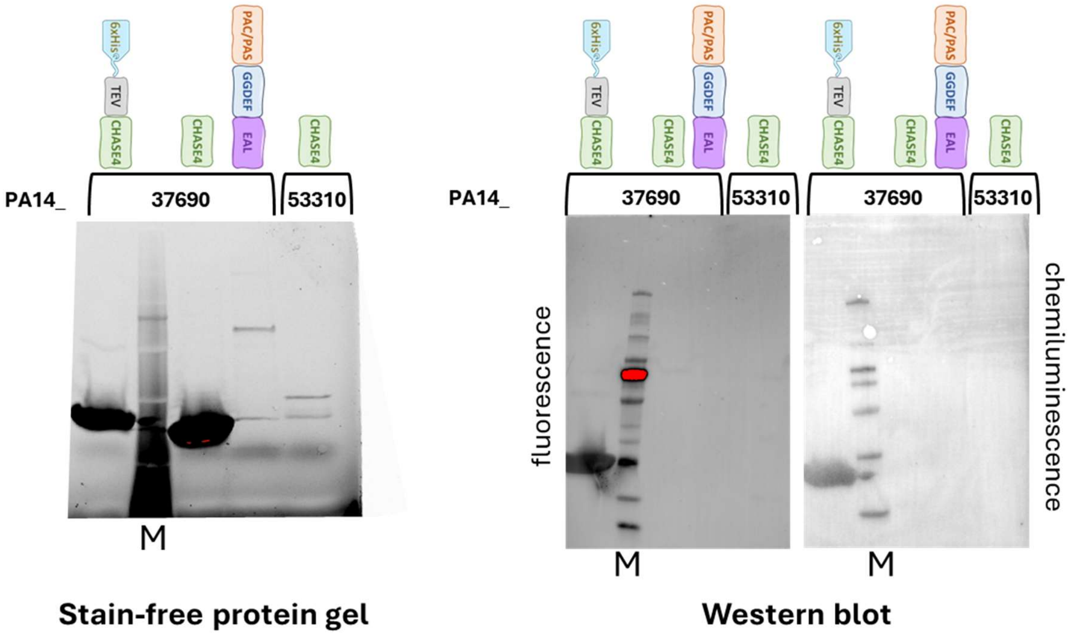

Figure S13. Western blot of His-tag free constructs using anti His-tag antibodies. To verify the proper His-tag removal of the constructs characterized in the present study, an SDS-PAGE and western blot analysis were carried out. Western blot was done in duplicate using two different secondary antibodies, allowing the fluorescence detection or the chemiluminescence-based detection (panel on the left). The Histag CHASE4 variant was used as positive control. Western blot confirms the proper removal of the His-tag tail by TEV-specific proteolysis.

Table SI. Thermodynamic signature of the interaction between CHASE4 PA14_37690 and copper ($CuSO_4$).

Variant	Number of site (n)	K_d (μM)	ΔH (kcal/mol)	-TΔS (kcal/mol)
**Wt**	1.22±0.03	1.27±0.30	-9.60±1.20	1.45±1.20
**H39A**	1.00±0.18	0.89±0.20	-3.90±0.80	-3.10±1.20

Table SII. Plasmids and protein expression conditions used in this study; all the constructs have been purchased.

Plasmid name	ORF	Competent cells used	Antibiotics (μg/ml)	Induction temperature and duration	Histag (N/C)
NcoI/XhoI cloning into pET-28a					
pET-28a 53310 FL	PA14_53310	BL21 (DE3) pLySs	kanamycin (30) + chloramphenicol (34)	T = 22°C t = 4 h	C
pET-28a 53310 CHASE4	PA14_53310 (57-314)	BL21 (DE3)	kanamycin (30)	T = 22°C t = 20 h	C
pET-28a 53310 TEV_CHASE4	PA14_53310 (57-314)	BL21 (DE3)	kanamycin (30)	T = 22°C t = 20 h	N
pET-28a 53310 Cyto	PA14_53310 (339-681)	BL21 (DE3)	kanamycin (30)	T = 16°C t = 20 h	C
pET-28a 37690 FL	PA14_37690 (1-860)	BL21 (DE3) pLySs	kanamycin (30) + chloramphenicol (34)	T = 22°C t = 4 h	C
pET-28a 37690 Cyto	PA14_37690 (274-864)	BL21 (DE3) pLySs	kanamycin (30) + chloramphenicol (34)	T = 22°C t = 4 h	C
pET-28a 37690 TEV_Cyto	PA14_37690 (274-864)	BL21 (DE3) pLySs	kanamycin (30) + chloramphenicol (34)	T = 22°C t = 4 h	N
pET-28a 37690 CHASE4	PA14_37690 (39-257)	BL21 (DE3) pLySs	kanamycin (30) + chloramphenicol (34)	T = 22°C t = 4 h	C
pET-28a 37690 TEV_CHASE4	PA14_37690 (39-257)	BL21 (DE3) pLySs	kanamycin (30) + chloramphenicol (34)	T = 22°C t = 4 h	N
NdeI/XhoI cloning into pET-28a					

pET-28a 53310 TEV_CHASE4	PA14_53310 (61-310) PAO1(117-364)	BL21 (DE3)	kanamycin (30)	T = 22°C t = 20 h	

Table SIII. Detailed protein purification protocols. Proteins harboring the cleavable N-terminal His-tag, TEV treatment and buffer exchange was done according to materials and methods.

Protein	Lysis buffer	Imidazole eluition (mM)	FPLC-column Superdex	Final buffer	MW (Da)	Predicted ε ($M^{-1}cm^{-1}$)
53310 FL	50 mM Hepes pH 7.6, 300 mM NaCl, 2.5% Glycerol, 1 mM PMSF	300	200 10/300 increase GL	50 mM Hepes pH 7.6, 300 mM NaCl, 2.5% Glycerol, 0.01% DDM	77860	66935
53310 CHASE4 (N/C terminal His-tag) (57-314)	50 mM Hepes pH 7.2, 300 mM NaCl, 1 mM PMSF)	200-300 (gradient)	75 increase 10/300 GL	50 mM Hepes pH 7.2, 300 mM NaCl	30754	29450
53310 TEV_CHASE4 (61-310)	50 mM Hepes pH 7.2, 300 mM NaCl, 1 mM PMSF)	insoluble	75 increase 10/300 GL	50 mM Hepes pH 7.2, 300 mM NaCl	31706	30940
53310 Cyto (C terminal His-tag)	50 mM Hepes pH 7.2, 300 mM NaCl, 1 mM PMSF)	300	None	50 mM Hepes pH 7.2, 300 mM NaCl	39611	24995
37690 FL	50 mM Hepes pH 7.6, 300 mM NaCl, 1 mM PMSF	300	None	50 mM Hepes pH 7.6, 300 mM NaCl, 0.01% DDM	95640	108665

37690 Cyto (N/C terminal His-tag)	50 mM Hepes pH 7.5, 300 mM NaCl, 1 mM PMSF	200	None	50 mM Hepes pH 7.5, 300 mM NaCl	67630	66871
37690 CHASE4/ Wt and H39A (N/C terminal His-tag)	50 mM Hepes pH 7.5, 300 mM NaCl, 1 mM PMSF	200 mM	75 increase 10/300 GL	50 mM Hepes pH 7.5, 300 mM NaCl	25039	29910

SUPPLEMENTARY METHODS.

PA14_53310 extraction with synthetic polymers. SMA2000 (Styrene Maleic Acid) and DIBMA Glycerol, HEPES (Cube Biotech) synthetic polymers were used.
Bacterial pellets of PA14_53310 were suspended in lysis buffer (50 mM Hepes pH 7.6, 300 mM NaCl, 2.5% Glycerol, 1 mM PMSF) and lysed by sonication on ice. Cell lysate was centrifuged 5' at 5000 rpm in order to remove any cellular debris. The supernatant was then ultracentrifuged at 35.000 rpm (~125*103 x g) for 1 h. The obtained pellet was suspended in buffer 50 mM Hepes pH 7.6, 300 mM NaCl, 5% Glycerol, 2.5% SMA2000 (Styrene Maleic Acid) or 50 mM Hepes pH 7.6, 300 mM NaCl, 5% Glycerol, 1.25% DIBMA Glycerol, HEPES (Cube Biotech), by using a homogenizer. The protein was then stirred at 4°C for 2 h to promote membrane bi-layer extraction, and was then diluited 1:1 with buffer 50 mM Hepes pH 7.6, 300 mM NaCl, 5% Glycerol and ultracentrifuged for 1 h at 27000 rpm. The obtained supernatant was then incubated at 4°C o.n. with 500 µl of Chelating Sepharose Fast Flow resin (GE Healthcare), previously equilibrated with buffer and nickel. The next day, the elution was carried out by increasing the imidazole concentration, with the protein eluting at 300 mM imidazole. Fractions containing the protein were analysed through SDS-PAGE and collected. Imidazole was removed with desalting column.

Membrane Scaffold Protein (MSP) nanodisc assembly. MSP1E3D1 construct was expressed and purified as described previously (Scribani Rossi et al., 2025) to produce the His-tag free membrane scaffold protein for nanodisc assembly. Solubilized POPC phospholipids (phosphatidylcholine, AVANTI polar lipids) were added to the PA14_53310 protein isolated in DDM detergent in a protein:MSP1E3D1:POPC ratio (1:5:300) and incubated 2 h at 4 °C. Detergent was removed as reported in (Scribani Rossi et al., 2025) and assembled nanaodisc were separated from empty nanodiscs by using Ni-IMAC, chelating Sepharose resin (GE Healthcare) pre-equilibrated with Ni^{2+} (by taking advantage of the His-tag tail present in the PA14_53310 protein). Nanodisc assembly has been carried out during DGC turnover, according to the materials and methods section, to favor the dimeric form of the transducer.

Western blot. After SDS-PAGE, the proteins were transferred onto nitrocellulose membranes (for chemiluminescence) and PVDF-LF (for fluorescence, Biorad) and

incubated overnight at 4 °C with the primary antibody: Anti-Histag 1:1000 (Bio-Rad). The following day the membranes were incubated with the secondary antibody anti-mouse 1:5000 (Santa Cruz Biotechnology) for chemiluminescence and anti-mouse 1:10000 (Bio-Rad, StarBright Blue 700)) for the fluorescence analysis for 1h at RT. Membranes were washed with PBS- 0.1% Tween 20 (chemiluminescence) and TBS casein 1% buffer (Fluorescence) and developed using either the chemiluminescence or the appropriate fluorescence channel at the system Chemidoc MP Imaging System (Bio-Rad).

REFERENCES.

Paiardini A, Mantoni F, Giardina G, Paone A, Janson G, Leoni L, Rampioni G, Cutruzzolà F, Rinaldo S. A novel bacterial l-arginine sensor controlling c-di-GMP levels in Pseudomonas aeruginosa. Proteins. 2018 Oct;86(10):1088-1096. doi: 10.1002/prot.25587. Epub 2018 Sep 8. PMID: 30040157.

Giardina G, Castiglione N, Caruso M, Cutruzzolà F, Rinaldo S. The Pseudomonas aeruginosa DNR transcription factor: light and shade of nitric oxide-sensing mechanisms. Biochem Soc Trans. 2011 Jan;39(1):294-8. doi: 10.1042/BST0390294. PMID: 21265791.

Scribani Rossi C, Parisi G, Paiardini A, Rinaldo S. Exploring Innovative Approaches to Isolate a One-Component c-di-GMP Transducer: A Pilot Study. Adv Exp Med Biol. 2025;1476:9-21. doi: 10.1007/5584_2023_787. PMID: 37608242.

Zhan X, Zhang K, Wang C, Fan Q, Tang X, Zhang X, Wang K, Fu Y, Liang H. A c-di-GMP signaling module controls responses to iron in Pseudomonas aeruginosa. Nat Commun. 2024 Feb 29;15(1):1860. doi: 10.1038/s41467-024-46149-3. Erratum in: Nat Commun. 2024 Oct 8;15(1):8707. doi: 10.1038/s41467-024-52012-2. PMID: 38424057; PMCID: PMC10904736.

Giardina G, Rinaldo S, Johnson KA, Di Matteo A, Brunori M, Cutruzzolà F. NO sensing in Pseudomonas aeruginosa: structure of the transcriptional regulator DNR. J Mol Biol. 2008 May 16;378(5):1002-15. doi: 10.1016/j.jmb.2008.03.013. Epub 2008 Mar 14. PMID: 18420222.

La Mar GN, Budd DL, Viscio DB, Smith KM, Langry KC. Proton nuclear magnetic resonance characterization of heme disorder in hemoproteins. Proc Natl Acad Sci U S A. 1978 Dec;75(12):5755-9. doi: 10.1073/pnas.75.12.5755. PMID: 282600; PMCID: PMC393052.

Golnak R, Xiao J, Atak K, Stevens JS, Gainar A, Schroeder SL, Aziz EF. Intermolecular bonding of hemin in solution and in solid state probed by N K-edge X-ray spectroscopies. Phys Chem Chem Phys. 2015 Nov 21;17(43):29000-6. doi: 10.1039/c5cp04529k. PMID: 26455378.
